# Virally mediated *Kcnq1* gene replacement therapy in the immature scala media restores hearing in a mouse model of human Jervell and Lange-Nielsen deafness syndrome

**DOI:** 10.15252/emmm.201404929

**Published:** 2015-06-17

**Authors:** Qing Chang, Jianjun Wang, Qi Li, Yeunjung Kim, Binfei Zhou, Yunfeng Wang, Huawei Li, Xi Lin

**Affiliations:** 1 Department of Otolaryngology, Emory University School of MedicineAtlanta, GA, USA; 2 Department of Otolaryngology-Head and Neck Surgery, Nanfang Hospital of Southern Medical UniversityGuangzhou, China; 3 Department of Otolaryngology, Eye & ENT Hospital, Fudan UniversityShanghai, China

**Keywords:** gene therapy, hearing restoration, Jervell and Lange-Nielsen syndrome, *Kcnq1* null mice, virus

## Abstract

Mutations in the potassium channel subunit *KCNQ1* cause the human severe congenital deafness Jervell and Lange-Nielsen (JLN) syndrome. We applied a gene therapy approach in a mouse model of JLN syndrome (*Kcnq1*^−/−^ mice) to prevent the development of deafness in the adult stage. A modified adeno-associated virus construct carrying a *Kcnq1* expression cassette was injected postnatally (P0–P2) into the endolymph, which resulted in *Kcnq1* expression in most cochlear marginal cells where native *Kcnq1* is exclusively expressed. We also found that extensive ectopic virally mediated *Kcnq1* transgene expression did not affect normal cochlear functions. Examination of cochlear morphology showed that the collapse of the Reissner’s membrane and degeneration of hair cells (HCs) and cells in the spiral ganglia were corrected in *Kcnq1*^−/−^ mice. Electrophysiological tests showed normal endocochlear potential in treated ears. In addition, auditory brainstem responses showed significant hearing preservation in the injected ears, ranging from 20 dB improvement to complete correction of the deafness phenotype. Our results demonstrate the first successful gene therapy treatment for gene defects specifically affecting the function of the stria vascularis, which is a major site affected by genetic mutations in inherited hearing loss.

## Introduction

Deafness caused by genetic mutations, which are responsible for more than half of all cases of congenital permanent hearing loss, has a prevalence of about 1–2 in every 1,000 human births (Smith *et al*, 2005). Genetic predisposition is also a significant factor in age-dependent hearing loss (ADHL) (Yamasoba *et al*, 2013), a major form of adult-onset sensorineural hearing loss affecting tens of millions of people (Dobie, 2008; Yamasoba *et al*, 2013). More than 100 deafness genes have been identified. Also, both our knowledge of the molecular etiology of deafness and our capability to diagnose genetic mutations have been greatly improved (Brownstein *et al*, 2011). Nevertheless, biological interventions based on cellular and molecular mechanisms that correct the root genetic causes of sensorineural hearing loss are not yet available. Currently, the major therapeutic options for sensorineural hearing loss are hearing aids and cochlear implant prostheses.

Most hereditary hearing loss is caused by homozygous recessive mutations (Lenz & Avraham, 2011; Shearer *et al*, 2013). The deafness genotype and phenotype relations usually are tightly defined (Smith *et al*, 2005). A monogenic mutation affecting the function of hair cells, supporting cells, or the stria vascularis (SV) are three major types of mutations causing severe hearing loss (Hilgert *et al*, 2009; Avraham & Kanaan, 2012). This means that most cases of genetic hearing loss are potentially amenable to gene replacement or augmentation therapy by exogenous expression of a single wild-type (WT) protein (Akil *et al*, 2012; Sacheli *et al*, 2012). Multiple research groups in the hearing field have worked for years to introduce gene therapy into clinical applications for the treatment of deafness. Experiments have repeatedly demonstrated that exogenous reporter genes such as green fluorescent protein (GFP) are expressed with high transduction efficiency by various types of viral vectors in the inner ear (Raphael *et al*, 1996; Sacheli *et al*, 2012). Although recent studies have yielded promising results with regard to the use of gene therapy to treat defective hair cells (Akil *et al*, 2012), work is still needed to demonstrate the efficacy of virally mediated gene therapy in treating more common genetic deafness resulting from mutations affecting the function of either supporting cells (e.g. those caused by mutations in *GJB2*) or cells in the SV.

Mutations in the *KCNQ1* gene (also known as *KvLQT1* or *Kv7.1*) are associated with Jervell and Lange-Nielsen (JNL) syndrome (Jervell & Lange-Nielsen, 1957), the phenotypes of which include congenital deafness and long QT intervals in the cardiogram, as well as sudden infant death syndrome and Romano–Ward syndrome (Lee *et al*, 2000). *KCNQ1* is widely expressed in cardiovascular muscle cells, the kidneys and stomach, and marginal cells in the SV of the inner ear. The *KCNQ1* is a voltage-gated potassium channel; its protein has 676 residues. The KCNQ1 consists of a cytosolic N-terminal domain followed by the S1-S4 voltage sensor, a canonical pore (S5-P-S6) domain, and a long cytosolic C-terminus. At least 16 mutations in the *KCNQ1* gene, typically recessive, cause JLN syndrome (Casimiro *et al*, 2001). The most common ones are missense mutations that result in single amino acid residue replacements. In the inner ear, *KCNQ1* co-assembles with *KCNE1* to play critical roles in the secretion of K^+^ into the endolymph and the establishment of the endocochlear potential (EP) (Lang *et al*, 2007).

**Figure 1:**
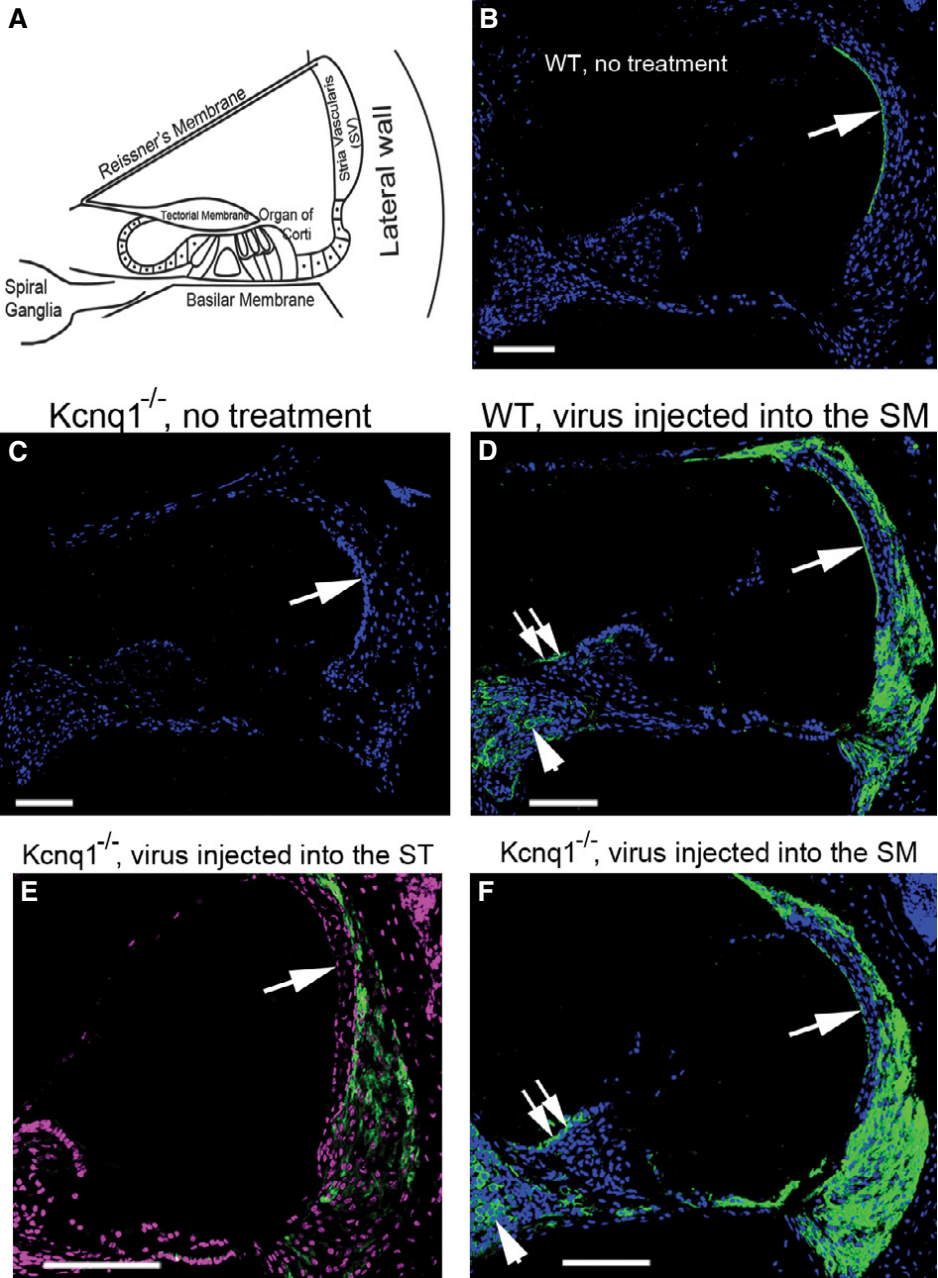
Cellular locations of native and virally mediated *Kcnq1* expression in the cochlea A Diagram showing the major landmarks of the cochlear section to facilitate comparison of data shown in (B–F).

The endolymphatic space in the cochlear duct is bound by epithelial cells of the membranous labyrinth on three sides by the Reissner’s membrane, the reticular lamina, and the lateral wall (Fig1A). *Kcnq1* is expressed exclusively in the apical membrane of the marginal cells in the SV. SV in the inner ear generates a high concentration of K^+^ in the endolymph and high extracellular endocochlear potential (EP, ∼+80 mV), both of which are crucial for the transduction of sound by HCs into neural signals (Lang *et al*, 2007). Inactivating the *Kcnq1* in mice produces a completely deaf model of human JLN syndrome, although the cardiac phenotypes are less prominent (Lee *et al*, 2000; Casimiro *et al*, 2001). In this study, we injected a *Kcnq1*-expressing AAV1 viral construct into the endolymph of *Kcnq1*^−/−^ mice in the early postnatal period. Results demonstrated for the first time that a gene therapy approach could be applied in a mouse model of JLN syndrome to successfully treat gene defects specifically affecting the functions of the SV.

B–F Immunolabeling results of *Kcnq1* (green) in cochlear cryosections are shown for uninjected WT (B), untreated *Kcnq1*^−/−^ (C), injected WT (D), and *Kcnq1*^−/−^ mice given injections into the ST (E) or SM (F). Cell nuclei were outlined by counterstaining with either DAPI (B–D, F) or Qnuclear deep red (E). Scale bars represent approximately 100 μm. Meaning of white arrows are given in the text.

## Results

### Viral inoculation into the scala media (SM) resulted in on-target and extensive ectopic Kcnq1 expression in the cochlea

Our immunolabeling data from WT cochlear sections (*n* = 5, see arrow in Fig1B) showing a thin green line at the border of the SV confirmed that *Kcnq1* is exclusively expressed by the marginal cells in the SV (Lang *et al*, 2007). The specificity of the immunoreactivity was supported by both the ultra-low background signal (Fig1B) and the disappearance of the thin line in the cochleae of *Kcnq1*^−/−^ mice (*n* = 6; see arrow in Fig1C). Since one of the aims of this study was to re-establish the missing *Kcnq1* expression (Fig1C) in as many marginal cells as possible, we first compared the viral inoculation efficiency achieved by injections into the SM and scala tympani (ST). For either *Kcnq1* (comparing panels E&F in Fig1) or green fluorescent protein (GFP) (comparing panels A & B in Supplementary Fig S1) expression, virus injection into the SM was the only route that resulted in successful transduction of the marginal cells, as indicated by a single arrow in Fig1D and F (and arrows in Supplementary Fig S1A). We never observed virally mediated *Kcnq1* expression (arrow in Fig1E) or GFP (arrow in Supplementary Fig S1B) in SV cells when injections were made into the ST (*n* = 5 in both cases).

These findings indicated that we need to inoculate viruses directly into the SM in order to re-establish missing *Kcnq1* expression (Fig1C) with high efficiency in the marginal cells. This choice of viral delivery route was also supported by our auditory brainstem response (ABR) test results, which showed that the correction of the deafness phenotype in *Kcnq1*^−/−^ mice was not achieved with viral inoculation into the ST (Supplementary Fig S1B).

The fact that native *Kcnq1* is found only at the apical membrane of the marginal cells (Fig1B; Lang *et al*, 2007) also facilitated our studies of virally mediated ectopic *Kcnq1* gene expression. When we compared immunolabeling results obtained in the injected cochlea of WT (Fig1D) and *Kcnq1*^−/−^ (Fig1F) mice, we found that *Kcnq1* was detectable not only in the marginal cells in the SV (see arrow, Fig1D and F), but also ectopically in cells in the lateral wall, spiral ganglia (arrowheads, Fig1D and F), and spiral limbus regions (double arrows, Fig1D and F). Importantly, ABRs obtained from WT mice injected with the *Kcnq1-*expressing viral construct (*n* = 6, Fig 5B, data points shown by filled circles) showed that ABR thresholds were indistinguishable from those of WT mice (*n* = 6, Fig 5B, data points shown with filled squares), indicating that strong, extensive ectopic *Kcnq1* expression (Fig1D) did not affect normal cochlear functions.

**Figure 2:**
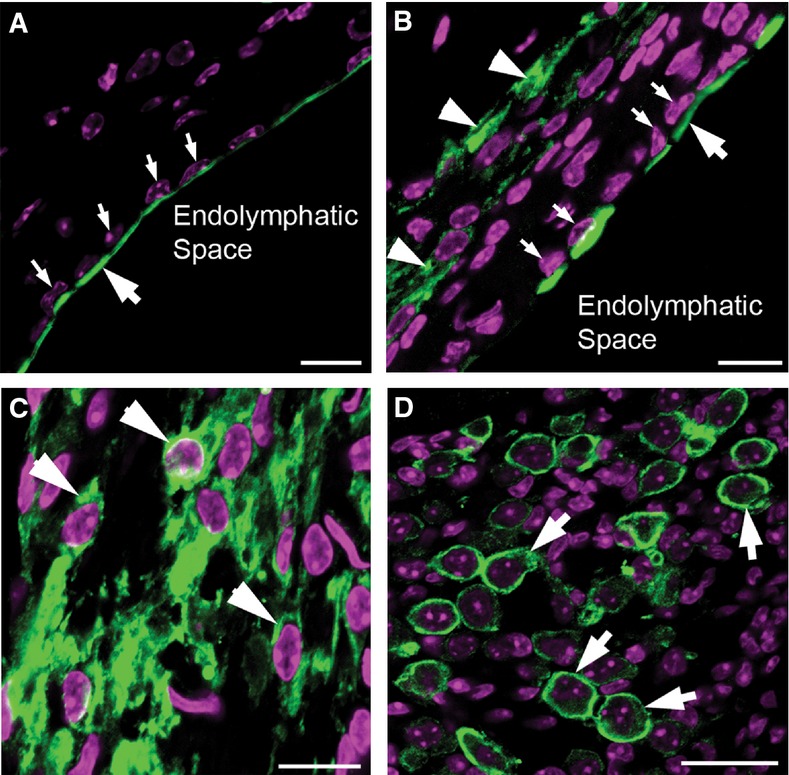
Polarized intracellular trafficking of native and virally expressed *Kcnq1* was found specifically in the marginal cells *Kcnq1* and cell nuclei were, respectively, labeled green and purple. The SV consists of three layers of cells; marginal cells are the first layer of cells on the side of the endolymphatic space. Cryosection through the SV of WT mice. The large arrow points to the native *Kcnq1* (labeled green) in WT mice. Smaller arrows show nuclei of the marginal cells.

*Kcnq1* is transported to apical side of the marginal cells (Lang *et al*, 2007). The polarized intracellular trafficking of native Kcnq1 protein was confirmed by our immunolabeling of WT cochlear sections (*n* = 5), as indicated by the big arrow in Fig2A (small arrows shows the locations of nuclei of the marginal cells). In the marginal cells of treated *Kcnq1*^−/−^ mice, similar polarized intracellular trafficking was observed for virally expressed Kcnq1 protein (in Fig2B, an example is indicated by a big arrow. Smaller arrows show the locations of nuclei of the marginal cells). In contrast, ectopically expressed *Kcnq1* in fibrocytes (arrowheads in Fig2B and C) and in the cells of spiral ganglia (Fig2D) showed prominent intracellular presence, but did not show polarized intracellular trafficking to any particular side of the cell membrane.

Cryosection through the SV of a *Kcnq1*^−/−^ mouse injected with AAV expressing *Kcnq1*. Labeled in green (bigger arrow) is the AAV1-expressed *Kcnq1*, found only in the apical membrane of the marginal cells. Smaller arrows show nuclei of the marginal cells. Arrowheads show *Kcnq1* immunolabeling in fibrocytes outside the SV.

Cryosection through the lateral wall of *Kcnq1*^−/−^ mice showing intracellular distribution of *Kcnq1* immunolabeling (green) in fibrocytes.

Cryosection through the spiral ganglia of cochlea of treated *Kcnq1*^−/−^ mice showing immunolabeling (green) in the cells of spiral ganglia (arrows). Data information: Purple staining in all panels is counterstaining with DAPI showing the locations of cell nuclei. Scale bars represent appropriately 50 μm.

**Figure 3:**
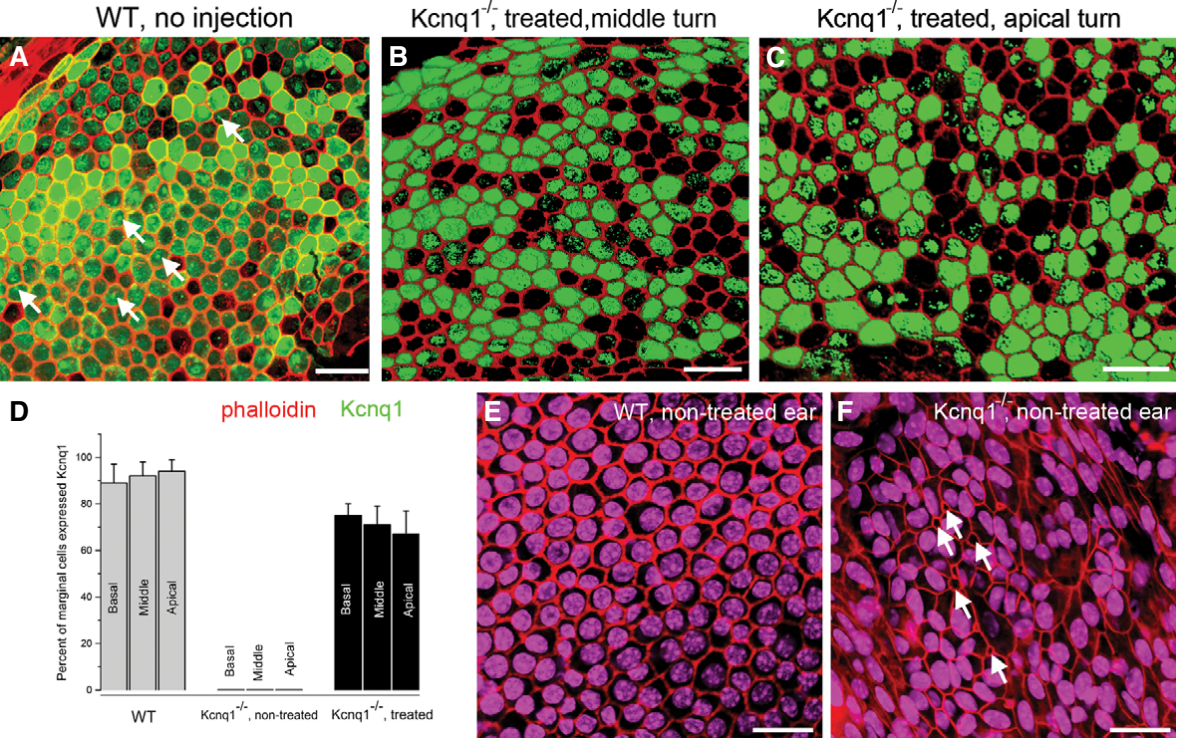
Quantification of *Kcnq1*-positive marginal cells and the effect of treatment on the cellular organization of the marginal cells in the SV The membranes of marginal cells are labeled (red) by phalloidin conjugated with rhodamine. A Immunolabeling results (*Kcnq1* labeled in green) in WT mice.

Immunolabeling using the flattened cochlear preparation (Chang *et al*, 2008) allowed us to quantify cellular *Kcnq1* expression (Fig3). The hexagonal cell membrane of individual marginal cells was outlined by labeling with phalloidin (labeled in red in Fig3). In the untreated cochleae of *Kcnq1*^−/−^ mice, we observed that the orderly hexagonal organization of the marginal cells (Fig3A–C,E) was damaged (Fig3F). We also found that, compared to the marginal cells in WT mice (Fig3E), the sizes of the marginal cells in untreated *Kcnq1*^−/−^ mice vary greatly. Moreover, many cells in untreated *Kcnq1*^−/−^ mice had missing nuclei (arrows in Fig3F), suggesting cellular degeneration or distress. Counting positively transduced marginal cells (Fig3B and C) yielded viral transduction efficiencies ranging from 75 ± 5% (*n* = 6) to 71 ± 8% (*n* = 6) to 61 ± 10% (*n* = 6) for marginal cells, respectively, located in the basal, middle, and apical turns (black bars on the right of Fig3D). These values were slightly lower than those in WT counterparts (Fig3D, gray bars), but were sufficient to prevent deafness (Fig 5B).

B, C Immunolabeling results (*Kcnq1* labeled in green) of treated *Kcnq1*^−/−^ mice, middle (B) and apical (C) turns, respectively.

D The percentage of marginal cells having positive *Kcnq1* immunolabeling signal above a visually detectable level is shown for WT (gray bars, left), untreated *Kcnq1*^−/−^ (middle), and treated *Kcnq1*^−/−^ mice (black bars, right). Data are given as mean ± SD (*n* = 6).

E, F The organization of marginal cells in the SV is outlined by labeling with phalloidin conjugated with rhodamine. Results from WT (E) and untreated *Kcnq1*^−/−^ mice (F) are compared. Data information: Scale bars represent approximately 50 μm.

Comparing the native *Kcnq1* (Fig3A) and virally mediated *Kcnq1* expressions (Fig3B and C), we found interesting similarities and differences:

- As in the cochlea of treated *Kcnq1*^−/−^ mice, native *Kcnq1* expression in a subgroup of marginal cells was always below the detection limit of immunolabeling in WT animals (Fig3A). The percentage of *Kcnq1* expression in the marginal cells was never 100% in both groups.
- Judging by immunolabeling intensity, we found that *Kcnq1* expression levels appeared to be more variable among WT marginal cells (Fig3A) than marginal cells of treated *Kcnq1*^−/−^ mice (Fig3B and C).
- Within individual marginal cells, virally mediated *Kcnq1* expression was more homogeneous. This was in sharp contrast to the many WT marginal cells that showed nonuniform or even spotted immunoreactivity (arrows, Fig3A), suggesting aggregation of the *Kcnq1* potassium channels in the cell membrane.

### Morphological and functional changes in the cochlea of *Kcnq1*^−/−^ mice in response to treatment

**Figure 4:**
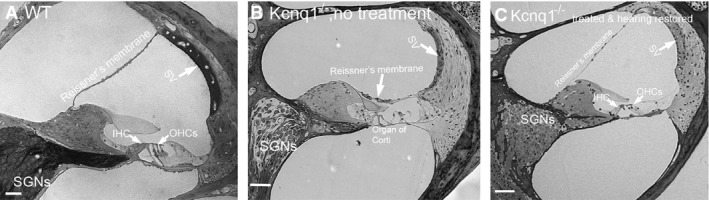
Comparison of cochlear morphology A–C Cochlear sections obtained from WT (A), nontreated *Kcnq1*^−/−^ (B), and treated *Kcnq1*^−/−^ mice (C) were compared. Major landmarks of the cochlear sections are labeled and pointed by arrows. Scale bars represent approximately 50 μm.

Consistent with previous reports (Lee *et al*, 2000; Casimiro *et al*, 2001), we found that untreated *Kcnq1*^−/−^ mice had a collapsed Reissner’s membrane, which was adherent to the spiral ligament and the tectorial membrane, resulting in disappearance of the SM. We also observed degeneration of inner and outer HCs, as well as supporting cells in the organ of Corti (compare Fig4A and B), and secondary degeneration of cells in the spiral ganglia (Fig4B). The mesothelial cells on the scala tympani side of the basilar membrane were absent as well. In treated *Kcnq1*^−/−^ cochleae, the collapse of the Reissner’s membrane was prevented (Fig4C), as was the death of hair cells, mesothelial cells, and cells in the spiral ganglia (Fig4C). EPs were 87.3 ± 5.7 (*n* = 5), 3.2 ± 0.2 (*n* = 5), and 85.3 ± 9.1 (*n* = 6) for WT, untreated *Kcnq1*^−/−^*,* and treated *Kcnq1*^−/−^ mice, respectively.

**Figure 5:**
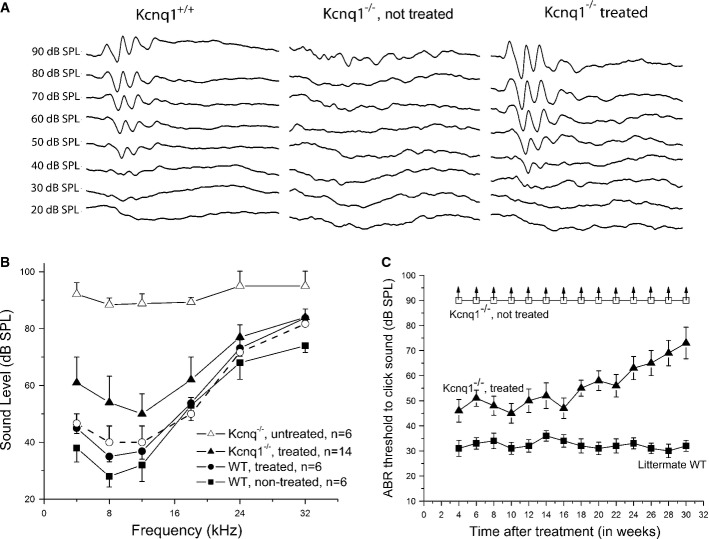
Comparison of the ABR data from WT, treated, and untreated *Kcnq1*^−/−^ mice Waveforms of ABRs are compared in WT, untreated *Kcnq1*^−/−^*,* and treated *Kcnq1*^−/−^ mice, as labeled above data traces. Series of averaged ABR data traces were evoked from tone-burst sounds with intensities ranging from 20 to 90 dB SPL.

Figure5A shows examples of ABR waveforms from the three groups of mice, and Fig5B and C shows summary of ABR thresholds. Similar ABR thresholds were obtained in WT mice treated or not treated by viral injection (Fig5B, n = 6, comparing data points given by filled squares and filled circles, none of the data points showed statistically significant differences on the Student’s *t*-test). Untreated *Kcnq1*^−/−^ mice had ABR thresholds around 90 dB SPL (*n* = 6, Fig5B; data points shown by open triangles). Injection of the *Kcnq1*-expressing viral construct into the endolymph of *Kcnq1*^−/−^ mice led to significant hearing preservation (*n* = 14, Fig5B; data shown by filled triangles). Differences between the average ABR thresholds obtained from treated and untreated mice were statistically significant (*P* < 0.05, Student’s *t*-test) for all frequencies tested between 4 and 32 kHz (Fig5B). Five of fourteen treated mice had ABR thresholds (Fig. 5B, Students’ *t*-test, dashed plot connecting open-circle data points) that were indistinguishable from those of treated WT mice (filled circles). In contrast, when the same viral construct was injected into the ST through the RW membrane (*n* = 7), no injected mice showed significant hearing improvement (Supplementary Fig S2B). We have repeated viral inoculations using solutions independently made from three batches. Data obtained by four different experimenters showed essentially the same results, suggesting that the treatment protocol used in this study yielded stable results (Supplementary Fig S2A).

Summary of averaged ABR thresholds at various frequencies for different groups of mice; untreated *Kcnq1*^−/−^ (open triangles), treated *Kcnq1*^−/−^ (filled triangles), injected WT (filled circles), and uninjected WT (filled squares). Plot legends are given in the figure. The plot with open circles connected with dashed lines represents average data from the five best cases of treated *Kcnq1*^−/−^ mice. Error bars represent standard error of the mean.

Click-evoked ABR thresholds of WT (filled squares), treated *Kcnq1*^−/−^ mice (filled triangles), untreated *Kcnq1*^−/−^ mice (open squares) measured 4–30 weeks after mice were born. Error bars represent standard error of the mean. Upward arrows indicate that click-ABR thresholds were at the maximal sound level that could be reliably measured by the system.

We examined the effect of hearing preservation in treated *Kcnq1*^−/−^ mice for up to 30 weeks (Fig5C). Click-ABR results (*n* = 5) demonstrated that the treatment effect was stable for the initial 18 weeks and then started to decline (Fig5C) at a rate of about 1.4 dB/week. By the end of 30 weeks, click-ABR thresholds in the treated mice increased by about 17 dB on average. However, the difference between treated and untreated ears was still statistically significant (Student’s *t*-test, *P* < 0.05). In addition, we found that even in *Kcnq1*^−/−^ mice that had worsening click-ABR thresholds (*n* = 3, data not shown), the gross cochlear morphology was normal for hair cells and cells in the spiral ganglia, as well as the position of the Reissner’s membrane.

## Discussion

Gene therapy studies in the hearing field have traditionally focused on the restoration of hearing by regenerating sensory hair cells (HCs) from surviving supporting cells, by using a cell replacement approach to restore normal cochlear function, or by expressing exogenous trophic factors (Raphael *et al*, 1996; Sacheli *et al*, 2012). Relatively few investigations have used genetically deaf mouse models to test treatment outcomes directly. Most studies have yielded either marginal hearing improvement (Maeda *et al*, 2009) or phenotype correction limited to morphological characteristics (Yu *et al*, 2013). Some successful cases have involved the use of technical approaches that are not directly applicable to humans (Maeda *et al*, 2005; Ahmad *et al*, 2007; Miwa *et al*, 2013). One exception has been a recent gene therapy study in which VGLUT3 knockout (KO) mice were treated (Akil *et al*, 2012). VGLUT3-expressing AAV1 was injected into the cochlea to replace the null VGLUT3. The treated VGLUT3 KO mice gained stable long-term hearing. When this study is compared to the current work, important similarities and differences emerge, as summarized in Table1. When reviewing this table, one important difference needs to bear in mind is that the cellular targets for treatment in the two studies are hair cells in the organ of Corti and marginal cells in the SV, respectively, which are two different sites in the cochlea.

**Table 1:** Comparison of the current study and a published study by Akil *et al* (2012)

	This study	Akil *et al*
Viral subtype	AAV1	AAV1
Promoter used	CBA	CBA
Virus injection time	P0–P2	P1–P12
Repeated with different batches of viral solution	Yes (Supplementary Fig S2)	Unclear
Targeted cells	Marginal cells	Inner HCs
Ectopic expression of therapeutic gene	Yes, and extensive	No
Ectopic expression of GFP	Yes	Yes
Long-term treatment effect	Deteriorated after 18 weeks	Maintained for at least 9 months
Intracellular trafficking	Located specifically to apical membrane of the marginal cells	Stayed uniformly and intracellularly in the inner HCs
% of expression in targeted cells	75 ± 5, 71 ± 8 and 61 ± 10% for marginal cells in the basal, middle, and apical turns, respectively	100% in the inner HCs through the cochlear turns
ST delivery for Trans-scala expression	Generally poor	100% inner HCs were transduced

It is interesting to note that although the same AAV subtype and promoter were used in both studies, our injections into the ST generally failed to transduce any cells lining the endolymphatic space (Supplementary Fig S1). This study also yielded a number of novel findings, among them the extensive ectopic expression shown by virally mediated *Kcnq1* in the cochlea (Figs1 and 2). These results are in contrast to virally expressed VGLUT3, which was exclusively in 100% of the inner HCs (Akil *et al*, 2012). Considering that other virally expressed exogenous proteins, including GFP (Akil *et al*, 2012; Wang *et al*, 2013) and connexin26 (Yu *et al*, 2013), all showed nonspecific expression, our results suggest that the cellular specificity achieved by VGLUT3 driven by a nonspecific promoter is an exception rather than a common phenomenon. The ectopic *Kcnq1* expression demonstrated in this work also gave us an opportunity to investigate possible side effects of such expression outside the targeted cells. Since ectopic *Kcnq1* expression in WT mice did not damage normal hearing (Fig5B, data points shown by filled circles), we conclude that *Kcnq1* expressed in the cells in the spiral ganglia, in fibrocytes of the lateral wall, and in interdental cells (Fig1D and F) probably did not form K^+^ channels that are harmful to the function of those cells. This is not surprising since it is known that functional potassium channels in the marginal cells need the co-assembly of *Kcnq1* and *Kcne1* (Lang *et al*, 2007). In addition to the lack of proper intercellular trafficking of *Kcnq1* to the cell membrane we have observed (Fig2), the expression of *Kcne1* may be lacking in ectopic cellular locations, thus preventing the formation of membrane channels.

Our results also showed for the first time that virally expressed exogenous protein was correctly trafficked intracellularly to its native membrane location (Fig2). *Kcnq1* encodes a potassium channel subunit that is known to be required for generation of the EP and the high K^+^ concentration, both of which are essential for auditory transduction (Barhanin *et al*, 1996; Sanguinetti *et al*, 1996). We found that exogenous *Kcnq1* was correctly targeted to these apical membranes; this was in sharp contrast to the diffuse intracellular distribution of virally expressed GFP or VGLUT3 in inner HCs (Akil *et al*, 2012). Interestingly, ectopically expressed connexin26 (Cx26) in the marginal cells is not transported to the cell membrane (Yu *et al*, 2013), but virally expressed Cx26 in supporting cells was correctly targeted to the cell membranes to form gap junctions (Yu *et al*, 2013). These findings suggest that crucial endogenous protein regulatory mechanisms govern the transportation and assembly of virally expressed proteins. These proteins are able to be trafficked like native proteins and co-assembled with their native molecular partners (e.g., Kcne1, Cx30) to form functional membrane channels.

One of the important tasks in conducting preclinical trials in animal models, assuming that the time course of disease progression and the phenotype characteristics observed in animal models can be applied in humans, is to examine reasonable boundary conditions for optimal treatment options in humans. This study has established a few of these boundary conditions for the treatment of *Kcnq1* null mutations:

1. We found that in order to have the hearing preserved in the *Kcnq1*^−/−^ mice, the percentage of marginal cells expressing the *Kcnq1* need not to be 100%. By immunolabeling criteria, the percentage of marginal cells expressed *Kcnq1* after viral injections is in the range of 61–75% (Fig3F). Whether higher transduction efficacy may give better or longer-lasting treatment effect is unknown.
2. Because of the collapse of the Reissner’s membrane and degeneration of the multiple types of cochlear cells observed in the mouse model (Fig4B), it appeared that the optimal timing for the treatment of the *Kcnq1* null mutation would be before these permanent histological changes happen. Any therapy implemented after malformation of the cochlea would be significantly more difficult. This finding may also serve as a guide to the treatment of other genetic deafness mutations that predominantly affect the morphological development of the cochlea.
3. With the viral type and promoter we tested in this study, it appears that the results of one-time treatment for mutations affecting the function of the SV are not permanent (Fig5C). Thus, either a new viral type must be tested or Supplementary treatment be done for longer-term efficacy.

Injections directly into scala media often produce side effects such as the breaking of cochlear structure or mixing of the perilymph and endolymph, both of which invariably lead to severe hearing loss (Kawamoto *et al*, 2001; Shibata *et al*, 2009). We found that injections into the perilymph did not give meaningful transduction in the marginal cells, while viral inoculation into the endolymphatic space after P5 almost certainly causes permanent hearing loss due to surgical procedures (Wang *et al*, 2013), thus negating the original purpose of the therapy. These results are consistent with those of most studies, which have shown that injection directly into the endolymph is needed to yield high transduction efficiency for cells lining the endolymphatic space (Fig1A) (Sacheli *et al*, 2012). A few authors have reported positive GFP labeling in the SV cells by injection into the ST. However, their results apparently lack the cellular resolution (Duan *et al*, 2002; Lei & Han, 2010) needed to determine whether or not marginal cells in the SV were transduced. By injecting in early postnatal (P0–P2) mice, when the cochlear bony shell is still soft, we avoided causing damage to cochlear morphology and to hearing sensitivities by the surgical procedures (Fig5B, plot connected by filled circles; Wang *et al*, 2013; Yu *et al*, 2013). The same surgical procedure would be difficult to apply to humans because the corresponding developmental period is embryonic. At that time, motivation to treat a condition that does not threaten life would not be high. Nonetheless, we have completed the first proof-of-principle study demonstrating that a gene therapy approach is effective for hearing preservation in a mouse model of gene defects specifically affecting the function of the SV. In the future, this approach may be used to test the feasibility of treating other inherited deafness cases in which the SV is the predominant site affected (e.g. mutations in *KCNE1*, *CCDC50, DFNA5, MYH14, TFCP2L3, TMPRSS3* genes).

Gene augmentation or replacement therapy for multiple inherited retinal degeneration diseases (e.g. Leber congenital amaurosis, choroideremia, Stargardt’s disease, and retinoschisis) has advanced to clinical phase I or phase II trials (Smith *et al*, 2012; Dalkara & Sahel, 2014). Our results in the auditory system suggest that as long as noninvasive gene delivery to the marginal cells in the scala media can be achieved in sufficient quantity and before the degeneration of cochlear cells, the human efficacy of such treatment suggested by the current study is optimistic. Recent advances in developing new viral vehicles aimed at effectively penetrating the blood–brain barrier (Foust *et al*, 2009; Manfredsson *et al*, 2009) or diffusing across dense tissue for gene delivery in the eyes (Dalkara *et al*, 2013) are promising candidates for further examining whether these methods could be used to treat genetic deafness in humans.

## Materials and Methods

### Preparation of viral constructs

Mouse *Kcnq1* cDNA was purchased from Open Biosystems (Pittsburgh, PA). SgfI and FseI restriction sites were added to the 5′ and 3′ ends, respectively, of the *Kcnq1* gene by a PCR-based procedure using primers: *Kcnq1*-F:5′-GCGATCGCATGGACACGGCCTCGT-3′ and *Kcnq1*-R: 5′-GGCCGGCCTCAGGAACCCTCATCAG-3′. The PCR product and the PCR II plasmid (Life Technologies, Grand Island, NY) were digested by SgfI and FseI. The *Kcnq1* was then incorporated into PCR II to form the PCR II-*Kcnq1* plasmid. An FseI restriction enzyme site was added to pENN.AAV.CB7.CI.RBG (Gene Therapy Program, University of Pennsylvania), using the primer: 5′-GGTACCGCGATCGCGTTTAAACGGCCGGCCCTCGAG-3′ to form the pAAV1-CB7-FseI plasmid. After digestion with EcoRI and FseI, *Kcnq1* was cloned into pAAV1-CB7-FseI to form pAAV1-CB7-*Kcnq1* plasmid. This plasmid was verified by restriction digestion and immunolabeling. We also used GFP-expressing viral constructs as controls; such constructs have been described in our publications (Wang *et al*, 2013; Yu *et al*, 2013).

Recombinant AAV particles were produced by double transfection of HEK293 cells with the AAV and AAV helper packaging plasmids pDP1rs expressing the AAV Rep2 and Cap1 genes (PlasmidFactory, Bielefeld, Germany). Recombinant AAV1 was harvested 72 h after transfection by three cycles of freezing and thawing. The crude viral lysate was then purified by fractionation with iodixanol-gradient centrifugation (Grieger *et al*, 2006). Viral genome copy titers were determined by quantitative PCR (Stratagene Mx3005p system, Agilent Technologies, Santa Clara, CA) using probes specific to the left inverted terminal repeat sequence of the AAV vector (Aurnhammer *et al*, 2012). The titer of AAV2/1 vector ranged from 5.0 × 10^12^ to 1.5 × 10^13^ genome copies/ml.

### Animal breeding, surgery, and virus injection procedures

Generation of *Kcnq1*^−/−^ mice (either sex) and genotyping procedures were described by Dr. Pfeifer’s group (Casimiro *et al*, 2001); he kindly provided mice for this study. Animal use protocols were approved by the Institutional Animal Care and Use Committee (IACUC) of Emory University. Heterozygous mice were bred to obtain *Kcnq1*^*+*/*+*^, *Kcnq1*^+/−^, and *Kcnq1*^−/−^ mice. Mice were divided into four groups (*N* > 6): WT controls; WT mice given viral injections into either the scala media or scala tympani; *Kcnq1*^−/−^ mice given viral injection into the scale media and used for ABR and cochlear morphological examinations; and *Kcnq1*^−/−^ mice given viral injection into the scale tympani and used for ABR and cochlear morphological examinations. The specific number of mice in each group is given in the Results. Mice were anesthetized by placing them on ice. An incision was made in the skin behind the ear to expose the otic bulla. The tympanic membrane and auditory ossicles were used as landmarks during surgery. The location of the basal cochlear turn was distinguished by its anatomical relation to the stapedius artery. WT and *Kcnq1*^−/−^ mice were injected in the left endolymphatic space with pAAV1-CB7-*Kcnq1* between postnatal day 0 (i.e. the day they were born, P0) and P2. The contralateral ear of the same mouse, which was used as a control, was either injected with pAAV1-CB7-*EGFP* or given no injection. The choice of viral subtype and the timing of injections at the early postnatal stage were based on our published results (Wang *et al*, 2013; Yu *et al*, 2013). The pAAV1-CB7-*Kcnq1* viral construct was confirmed by *in vitro* transfection of HEK293 cells, which showed that 100% of the cells in cultures were transfected *in vitro*. Each injection took about 10 min to complete. The surgery protocol was approved by the Emory IACUC protocol.

Viruses used in injections were resuspended in 0.01 M phosphate buffer. Injection of a small amount of fluid was done using a Picospritzer III pressure microinjection system (Picospritzer III; Parker Hannifin, NY). The pressure source was an air tank regulated at an output pressure of 20 psi. Glass micropipettes with a tip size of 10–15 μm were made on a P-2000 horizontal pipette puller (Sutter Instrument, Novato, CA), back-filled with viral solution, and controlled by a micromanipulator (MP-285, Sutter Instrument, Novato, CA). The glass micropipettes were controlled to penetrate into either the scala media through the soft bony cochlear shell of early postnatal mice near the basal cochlear turn or the scala tympani through the round window membrane. We ejected approximately 0.5 μl of fluid out of the tip of glass pipettes by controlling the duration (12 ms) and the number of pressure pulses (set at 12). Fast green dye (Sigma-Aldrich, St Louis, MO), which is visible under bright-field illumination with a dissecting microscope (Stemi2000; Carl Zeiss, Oberkochen, Germany), was included in the solution to help visually confirm fluid ejection. After surgery, mice were allowed to recover on a 37°C heating pad (model TR-100, Fine Science Tool Inc., Foster City, CA) before returning to the animal housing facility. More details of surgical and injection procedures have been given previously (Wang *et al*, 2013; Yu *et al*, 2013).

### Examination of cochlear morphology and immunolabeling of Kcnq1 expression

Anesthetized animals were perfused via cardiac catheter, first with 10 ml 1xPBS and then with 15 ml of a mixture of 2% paraformaldehyde and 2% glutaraldehyde. The experimenters were unaware of the genotype of the mice. After removal of the temporal bone, the inner ear was fixed in 4% PFA overnight at 4°C and decalcified in 10% EDTA for 3 days at 4°C. The tissues were postfixed with 1% osmium tetroxide for 1 h, dehydrated serially in 30, 50, 70, 95 and 100% ethanol, and then embedded in epoxy resin (Ted Pella Inc., Redding, CA). Cochlear sections were cut and stained with toluidine blue according to our previously published protocol (Sun *et al*, 2009). Immunolabeling was done using either cochlear cryosections or whole-mount preparations (Chang *et al*, 2008) of the SVs of adult mice (∼45 days after birth). Dissected cochlear samples were fixed in 4% paraformaldehyde for 20 min, permeabilized in 0.1% triton in phosphate-buffered saline (PBS) for 30 min, and blocked in 10% goat serum in PBS for 1 h. Primary antibodies against *Kcnq1* (Santa Cruz Biotechnology, Dallas, TX) were labeled first at 4°C overnight. The specificity of the *Kcnq1* antibody was examined by Western blotting. Only a single band was observed on the gel (data not shown). After washing three times in PBS, samples were incubated with Alexa 488-conjugated secondary antibody for 1 h at room temperature. Counterstaining for cell nuclei was done with either 4′,6-diamidino-2-phenylindole (DAPI) or Qnuclear deep red (both from Life Technologies, Grand Island, NY). Cell membrane was stained with isothiocyanate-conjugated phalloidin (Sigma-Aldrich, St. Louis, MO). Samples were then mounted in fluoromount-G antifading solution and examined under a Zeiss LSM 510 confocal microscope. To quantitatively assess gene transfection efficiency in the marginal cells, we calculated the percentage of transduced cells by counting the number of *Kcnq1*-positive cells (Fig3) and then divided the number by the total number of marginal cells in the field of view. More details of the immunolabeling protocol have been described previously (Ahmad *et al*, 2007; Chang *et al*, 2008).

### Functional assays for measuring ABRs, the EP, and vestibular responses

After viral inoculations, we measured ABRs in adult mice at time points given in the Results. Tone-burst ABR is an objective measure of the hearing threshold at specific frequencies. The ABR testers were unaware of the genotype of the mice. We presented sound stimuli to mice to test frequency-specific hearing thresholds at 4k-32k Hz (Fig5). Click ABR was also used in long-term follow-up studies. Data were given as mean ± standard error of the mean (mean ± s.e.). During ABR tests, we anesthetized mice with a mixture of ketamine hydrochloride (80 mg/kg) and xylazine (10 mg/kg). A plastic tube connected to the speaker was inserted into one ear to deliver sound stimuli generated by the BioSig software package (Tucker-Davis Technologies, Alachua, FL). The contralateral (uninjected) ear was also tested as a control. Details of the ABR and EP testing methods are given in our published papers (Ahmad *et al*, 2007). We monitored circling behavior, head tilt, and swimming ability of the injected mice; these parameters reflect vestibular functions.

## Supporting Information

**Supplementary Figures**

**Review Process File**
