# Supplementary material for: Virally mediated Kcnq1 gene replacement therapy in the immature scala media restores hearing in a mouse model of human Jervell and Lange-Nielsen deafness syndrome
Source: EMBO Mol Med. 2015 Jun 17;7(8):1077–86. doi: 10.15252/emmm.201404929 (PMC4551345; doi:10.15252/emmm.201404929)

Table of Contents, supplemental material

1) Supplemental figure 1.
2) Supplemental figure 2.

Supplemental Figure 1.
Virally mediated GFP expression in cochlea obtained by two different injection routes. (A) Results obtained by injection into the SM and (B) ST of WT mice are compared. Three arrows in (A) point to GFP-positive cells inside the SV. Small arrows in (B) point to GFP-positive cells in the lateral wall. One bigger arrow in (B) points to the location of the SV in cochlear section. Cell nuclei were outlined by counterstaining with Qnuclear deep red. Scale bars represent approximately 100 µm.

Supplemental Figure 2.
(A) Unaveraged ABR thresholds measured from individual animals. Different symbols represent results obtained by four different experimenters. Filled symbols are ABR results obtained from untreated Kcnq1-/- mice. (B) ABR thresholds obtained from Kcnq1-/- mice received viral injection into the scala tympani.

Supplemental Figure1

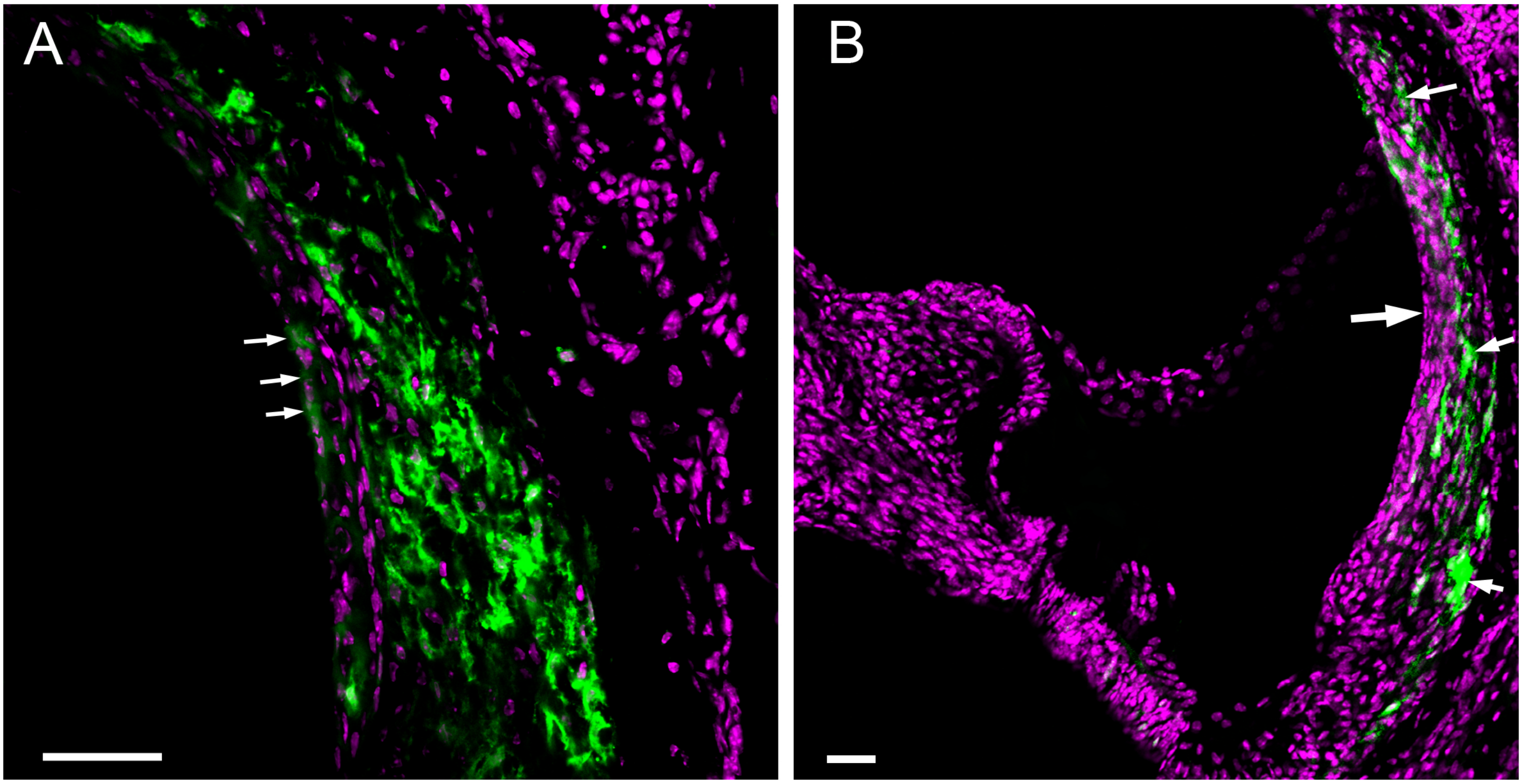

Supplemental Figure 2

A)

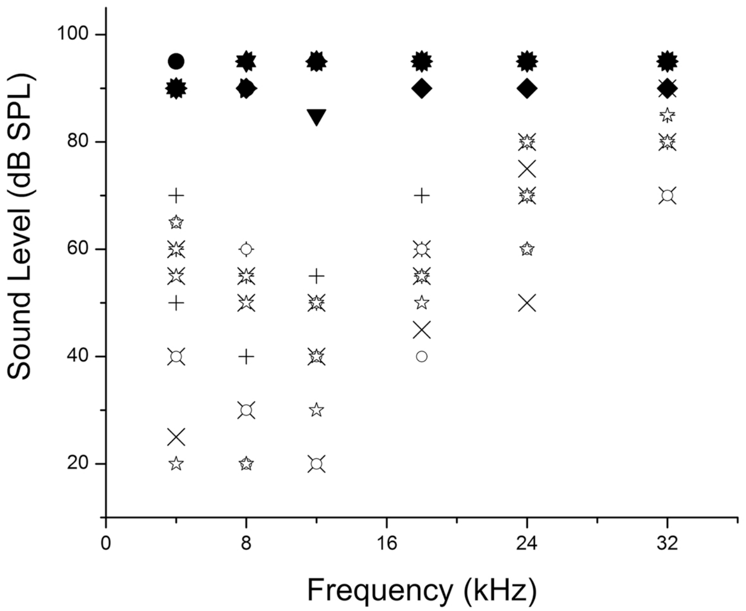

B)

Tone-burst ABR Thresholds
measure from mice injected
with viral solution into the scala tympani

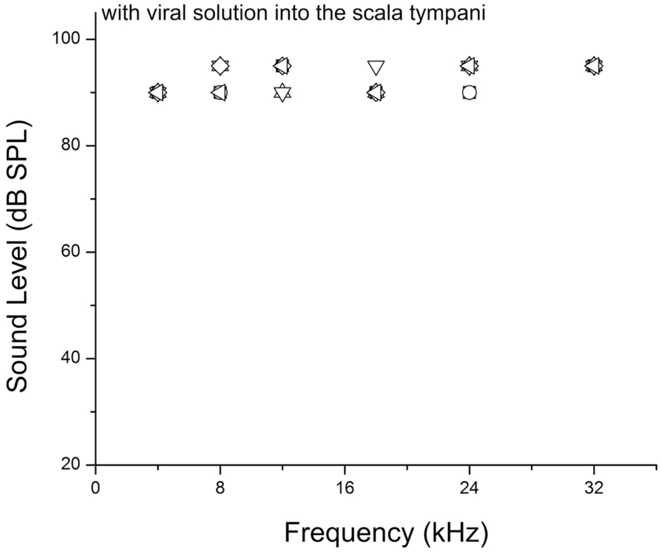

Supplement: Supplementary file 1 [file emmm0007-1077-sd1.pdf]
