# Supplementary material for: Virally mediated Kcnq1 gene replacement therapy in the immature scala media restores hearing in a mouse model of human Jervell and Lange-Nielsen deafness syndrome
Source: EMBO Mol Med. 2015 Jun 17;7(8):1077–86. doi: 10.15252/emmm.201404929 (PMC4551345; doi:10.15252/emmm.201404929)
Supplement: Supplementary file 2 [file emmm0007-1077-sd2.pdf]

EMBO
Molecular Medicine

Virally mediated *Kcnq1* gene replacement therapy in the immature scala media restores hearing in a mouse model of human Jervell and Lange-Nielsen deafness syndrome

Qing Chang, Jianjun Wang, Qi Li, Yeunjung Kim, Binfei Zhou, Yunfeng Wang, Huawei Li, Xi Lin

Corresponding author: Xi Lin, Emory University School of Medicine

---

Review timeline:

Submission date:	04 December 2014
Editorial Decision:	09 January 2015
Revision received:	21 March 2015
Editorial Decision:	15 May 2015
Revision received:	17 May 2015
Accepted:	21 May 2015

Transaction Report:

(Note: With the exception of the correction of typographical or spelling errors that could be a source of ambiguity, letters and reports are not edited. The original formatting of letters and referee reports may not be reflected in this compilation.)

Editor: Roberto Buccione

1st Editorial Decision 09 January 2015

Thank you for the submission of your manuscript to EMBO Molecular Medicine. We have now heard back from the three Reviewers whom we asked to evaluate your manuscript.

You will see that the Reviewers are supportive of your work, although they do mention a number of partly overlapping issues. This prevents us from considering publication at this stage. I will not dwell into much detail, as the evaluations are detailed and self-explanatory. I would like, however, to highlight the main point of concern.

All three Reviewers note that your work outlines a strategy designed to prevent the development of the syndrome, rather than to cure/treat it. This, in addition to suggesting a careful revision of the wording throughout the manuscript (including the title, which is currently misleading), also raises a potential ethical issue. In fact, the treatment would have to be administered to infants rather than adults; the ethical concerns associated with treating infants with a complicated procedure for a non life-threatening disease might be high. These aspects must be considered and thoroughly discussed in your revision.

Reviewer 1 lists a number of important issues for you to act upon and among these, touches upon the global clinical relevance of your work and potential transferability of your approach to adults. S/he would also like (as Reviewer 3) more details on the surgical procedure.

Reviewer 2 asks for many important clarifications on presentation, methods and conclusions that appear reasonable and approachable and therefore will require your action.

© EMBO

Reviewer 3 is perhaps more reserved, but still positive. S/he provides many opportunities for improvement that I fully agree with.

Considered all the above, while publication of the paper cannot be considered at this stage, we would be pleased to consider a revised submission, with the understanding that the Reviewers' concerns must be addressed as outlined above, with additional experimental data where appropriate and that acceptance of the manuscript will entail a second round of review.

Please note that it is EMBO Molecular Medicine policy to allow a single round of revision only and that, therefore, acceptance or rejection of the manuscript will depend on the completeness of your responses included in the next, final version of the manuscript.

EMBO Molecular Medicine now requires a complete author checklist (http://embomolmed.embopress.org/authorguide#editorial3) to be submitted with all revised manuscripts. Provision of the author checklist is mandatory at revision stage; The checklist is designed to enhance and standardize reporting of key information in research papers and to support reanalysis and repetition of experiments by the community. The list covers key information for figure panels and captions and focuses on statistics, the reporting of reagents, animal models and human subject-derived data, as well as guidance to optimise data accessibility.

As you know, EMBO Molecular Medicine has a "scooping protection" policy, whereby similar findings that are published by others during review or revision are not a criterion for rejection. However, I do ask you to get in touch with us after three months if you have not completed your revision, to update us on the status. Please also contact us as soon as possible if similar work is published elsewhere.

I look forward to seeing your revised manuscript in due time.

***** Reviewer's comments *****

Referee #1 (Remarks):

The paper "Virally-mediated gene therapy restores hearing in a mouse model of human Javell and Lange-Nielsen syndrome" presents a set of elegant experiments demonstrating the outcome of gene transfer of the Kcnq1 gene into ears of mutant mice. The results show that treatment, when administered in developing ears, can rescue the morphology and the function in these ears, when they mature. These are novel and exciting results that help advance the protocols for inner ear therapy for cases of genetic deafness, where no biological treatment is now available.
Several changes are necessary to enhance the quality of the presentation.
What's the meaning of: Moreover, we have obtained consistent therapeutic effects early...
The work is done in immature ears. This needs to be clear from the onset, and clearly mentioned in the abstract and perhaps also in the title. Along this line: it should be good to mention / speculate (Discussion) what it would take to accomplish this in mature ears.
The entire first paragraph is dedicated to what was done or not done by others, as prelude for the current data. It would be more elegant to be informative about the specific mutation in humans, the mouse, the approach... etc.
The introduction needs more detail about Kcnq1, the gene family, structure, function, gene roles in other tissues, other mutations, and more on the human phenotype. The fact that the human mutation is very rare should be mentioned and it would not deter from the enthusiasm for the results.
The second paragraph (line 60) jumps from topics, needs focus.
Correct this sentence for style: Kcnq1 is exclusively expressed by the marginal cells in the SV, where the high concentration of K+ in the endolymph and high extracellular endolymphatic potential (EP, ~+80 mV) are generated, and both are crucial for the 70 transduction of sound by hair cells into neural signals...
line 71 produced a mouse model
line 76: These results demonstrated for the first time
line 82 expression
Line 84: the EL space is bordered by epithelial cells of the membranous labyrinth, not by the basilar

membrane
The Results section should not start with Introduction material
Fig 1 D - F: re SGN expression, the observation is probably correct, but expression may also possibly be in Schwann cell, so a higher magnification inset may help clarify that.
Line 126 suggesting signs of cellular degeneration... not sure unless a later stage is done. Clearly, the cell borders and junctions are not normally organized. Also: since phalloidin mostly stains the adherens junction, it is nice for delineating cell-cell borders but does not reveal much about the tight junctions. The authors have the expertise for staining the latter, which might shed light on the pathology.
Line 134: move discussion items to discussion
A better explanation is needed regarding why GFP is in the cytoplasm and Kcnq1 in the membrane.
The changes in Reissner's membrane are striking. I am not sure if the term collapse is appropriate. I see the membrane inserting into the lower rather than upper part of the lateral wall. Has this been described before in any other pathology, genetic or environmental? With such anatomy, if the phenotypic rescue is attempted at P21 and the SV starts to function, would perilymph become endolymph like?
I also see in Fig. 4B that mesothelial cells in the ST side of the BM are absent. Perhaps it would be useful to point this out. Their absence is reversed with the treatment.
Line 168: the argument about "stable results" is unclear. The take-home message I get from the data is that in this model SM injections are needed for reaching cells of the membranous labyrinth, which has been shown before using Ad but has not yet been well characterized using an AAV. Also, these results are not in agreement with the Akil data on Vglut3, a fact that could be mentioned and discussed.
The Discussion organization, tone and focus need to change. It now focuses on advances made by the authors and on clinical applicability. In reality, clinical applicability is very remote because current data are on developing ears in "ear age" equivalent to roughly mid-gestation in humans. Also, SM access is currently difficult to impossible, at any age. Instead, I suggest the focus should be on cell biology of KCNq1, its role in the SV, why its presence in other ear cell types does not degrade thresholds, and why hearing is not better than normal and EP not higher than normal when levels of KCNQ1 are increased beyond normal. Discussion of the technique and choice of vector is also interesting and relevant (some of this is included, but some expansion may be useful). Best to conclude with the clinical applicability (or lack thereof) and what it would take to perform this on mature ears and accomplish rescue in the relevant model system and in human.
The Li citation should probably be Dual et al. (first name Mao Li). I did not check other citations.
Line 298: were authors approved in their animal use protocol to operate on both ears of the P 0-2 mouse? At same session? More details are needed.
Histology was done at ages 1 - 3 months and physiology 1-2. It would be helpful to know how many of the animals were processed at the later time points, which would make the rescue outcome more exciting.
The Impact section should be revised as the rest of the document is revised based on the comments.
Some of the figure legends need to include a little more detail to orient non-experts (in inner ear histology) around the tissue.

Referee #2 (Comments on Novelty/Model System):

The authors present a very interesting early postnatal Approach that successfully restores deafness based on Kcnq1 deficiency. Early postnatal or neonatal strategies are also for other kind of diseases a very important information source to study underlying mechanisms and set up new innovative strategies. However, human clinical neonatal treatment is much more critical, especially if it is not addressing a life threatening disease. With regards to deafness, the socio-economical impact is clearly lying on adult elderly patients. Therefore, I counted the medical impact 'only' as medium.

© EMBO

Referee #2 (Remarks):

Extended life time of the steadily expanding human population places deafness to one of the major future challenges for public health care systems worldwide with high social-economical impact. Though cochlear implants have proven their effectivity in the clinics, they are not suitable for all deafness patients. Therefore, novel innovative strategies based on gene and cell therapy may complement implant technologies or even substitute them. These new strategies are currently on their way to their translation into the clinics. Promising results in the transduction of hair cells have been achieved with adenoviral and adeno-associated viral vectors (AAV). As such, Akil et al. could recently reach a 100% transduction efficiency of the target inner hair cells and successful restoration of the Vglut3 deficiency when using an AAV1 vector. Remarkably, Vglut3 was found to be expressed only in the target cell population, although a non-specific promoter was used to express the transgene.

Here, Chang Q and colleagues present a preclinical AAV2/1 gene therapy approach to restore hearing of the Javell and Lange Nielsen syndrome (defect of the potassium channel gene Kcnq1). Treating Kcnq1 knock-out mice in the early postnatal phase, they could reach impressive results: restoration of the cochlear morphology, regain of endolymphatic potential and auditory brainstem response. These results are even more remarkable, as JLN syndrome represents a hearing defect that affects the function of the stria vascularis. Nevertheless, there are several obstacles within the presented manuscript that have to be clarified:

1) Clear presentation of the experimental setup and the related results is not good. This includes:
- size of the different mouse cohorts used (including positive and negative controls)
- which timepoint was exactly used for which mouse (cohort) in terms of vector administration (P0 or P2?) and harvest (1 month or 3 months?)
- which mouse received which vector dosage? Any differences in the outcome?
- Was the Kcnq1 transgene codon optimized?
Therefore, an additional supplementary table addressing this point is advisable.
2) The main point/advance of the presented data is the correction of genetic defects that affect the stria vascularis (SV). Repetitive experiments to prove the reliability of the obtained results is therefore decisive. Thus, it is unclear why the authors do not show the important 'negative' results of their injections in the scala tympani (ST) through the round window membrane and associated repetitions for SV and ST injections performed by different researchers or, better said, independent experiments, respectively (page 7, lines 163 - 168).
3) There are kind of diverging results by the recent Akil et al. study and the data presented here (page 10, lines 225 - 231). Please discuss this aspect in more detail, including AAV serotypes and dosages used, animal age at infection and time of sacrifice etc.
4) Very important, as the authors outline in their last paragraph of the discussion section, is the longterm effect of hearing restoration beyond the 1 -3 months study points in this manuscript. Did the authors not have any additional treated animals under investigation for longterm studies, at least to give a hint whether efficiency without side effects remains stable?
5) The authors should comment on the clinical translation opportunity of their early postnatal approach. This approach will not work for adult mice and for humans, right? The route to go will then be directly via the perilymph, rather than the scala media?
6) Each abbreviation has to be tendered before use. Please recheck your manuscript, e.g. QT, ST.

Referee #3 (Comments on Novelty/Model System):

1. Medium because there are a number of presentational issues.
2. High because any gene therapy approach that seems to work in the inner ear is novel, and the functional "rescue" (ABR thresholds) is striking.
3. Medium because it would be necessary to transfect the cochlear duct long before birth in humans with mutations in this gene, so it is more an avoidance of the development of the deafness than a treatment for the deafness.

4. The model, a mouse knockout of the orthologous gene, replicates many of the auditory features of the human disease.

The only ethical issue is the amount of surgery required to inject the cochlea, which is not described in the current text at all.

Referee #3 (Remarks):

This paper reports a convincing experiment where injection of a Kcnq1-containing AAV into the endolymph of the cochlea in Kcnq1 knockout mice leads to expression of the gene in marginal cells of the stria vascularis and avoidance of the expected pathology, such as raised ABR thresholds, collapse of Reissner's membrane and degeneration of sensory hair cells. Injection into scala media was successful in largely rescuing the hearing loss, while injection into scala tympani was not effective. Extensive ectopic expression was observed, but this did not appear to have any clear adverse effect. The limitation of the findings is that the injection was carried out at a very immature stage of development, before the normal start of full strial function in producing the highly-positive endocochlear potential in scala media. Therefore, the finding suggests that the pathology can be avoided before it begins by transfection with the AAV, but not that the deafness can be treated using this approach. Nonetheless, this is an important and interesting report. The presentation can be improved in several ways.
Title, abstract, and throughout the text: the human disease is Jervell and Lange-Nielsen, not Javell.
Abstract, line 26: The gene therapy does not treat the deafness phenotype, but instead prevents deafness from developing, so this sentence needs revising.
Abstract line 32: The potential is strictly the endocochlear potential, not endolymphatic potential, because not all endolymph-filled chambers have this high potential.
Introduction, line 74: The treatment does not restore normal cochlear morphology and function, but instead prevents the pathology from developing, so this sentence needs to be revised.
Results, line 84: The physiological boundary of scala media and endolymph is the reticular lamina - the upper surface of the hair cells and surrounding supporting cells, not the basilar membrane which is relatively leaky.
Results, line 93: Define SM and ST when first used.
Results; line 104-105: Say what this comparison reveals, rather than leave it to the reader to guess what the difference is.
Results, line 134: In point (1), clarify whether you refer to native Kcnq1 expression, or expression from the transgene.
Results, line 149: There is no evidence presented that Reissner's membrane has returned to its WT position, because it is most likely given the timing of the injections that the collapse of this membrane was avoided, so this needs rewording.
Discussion, line 215: This is the first time that the timing of the injection has been specified, so it would be helpful to the readers to give the stage of injection much earlier in the main text to prevent misunderstanding.
Discussion, lines 236-237: Even though the surgical technique has been described in a previous paper, it would be very helpful to give a brief outline in this paper (either here or in the methods) so that the implications for gene therapy can more easily be appreciated by the readers.
Discussion, line 259: The data for three different batches of viral solutions was not presented, so this conclusion is not supported by the results presented. Maybe the ABR thresholds for individual mice could be presented with batch number indicate by different symbols in the ABR threshold figure?
Methods, line 303-304: Say briefly what the exposure method involved.
Methods, line 305: Was artificial endolymph used when injecting into scala tympani, rather than artificial perilymph?
Figures: It appears that the scale bars are incorrect (too short for the distance given) for all figures. Please check.
Fig 2 legend, B, line 518: Clarify whether this mouse was injected or not.
Fig 3 legend, line 528 and 529: These appear to be whole mounts not sections, so remove the words "section".
Expanded view, Fig 1: Is this a supplementary figure? It does not look like an expanded view of Fig 1. State whether the samples are from mutants or WT mice.

1st Revision - authors' response 21 March 2015

Referee #1:

What's the meaning of: Moreover, we have obtained consistent therapeutic effects early...

------Answer: We have modified the abstract, the sentence "Moreover, we have obtained consistent therapeutic effects ……" has been removed. In addition, we have presented unaveraged ABR data measured by different experimenters (Supplemental Fig. 2A) to show that we have obtained consistent therapeutic effects.

Modified abstract is copied here:

"Mutations in the potassium channel subunit *KCNQ1* cause human Jervell and Lange-Nielsen (JLN) syndrome with a severe congenital deafness. We used the gene therapy tested in a mouse model of JLN syndrome (*Kcnq1*$^{-/-}$ mice) to prevent the deafness phenotype from developing in the adult stage. A modified Adeno-associated virus construct carrying a *Kcnq1* expression cassette was injected postnatally (P0-P2) into the endolymph, which resulted in *Kcnq1* expression in most cochlear marginal cells where the native *Kcnq1* is exclusively expressed. We also found that extensive ectopic virally-mediated *Kcnq1* transgene expression did not affect normal cochlear functions. Examinations of cochlear morphology showed that the collapse of the Reissner's membrane, degeneration of hair cells and cells in the spiral ganglia were corrected in *Kcnq1*$^{-/-}$ mice. Electrophysiological tests showed normal endocochlear potential in treated ears. In addition, auditory brainstem responses showed significant hearing preservation in the injected ears, ranging from 20 dB improvement to complete correction of the deafness phenotype. Results demonstrated the first successful gene therapy treatment for gene defects specifically affecting the function of the stria vascularis, which is a major site affected by genetic mutations in inherited hearing loss cases."

The work is done in immature ears. This needs to be clear from the onset, and clearly mentioned in the abstract and perhaps also in the title. Along this line: it should be good to mention / speculate (Discussion) what it would take to accomplish this in mature ears.

------Answer: We have changed the title to "Virally-mediated expression of *Kcnq1* in the immature scala media corrected the deafness phenotype in a mouse model of human Jervell and Lange-Nielsen syndrome".

In abstract (starting from page1 line25), we changed the second sentence to "We used the gene therapy tested in a mouse model of JLN syndrome (*Kcnq1*$^{-/-}$ mice) to prevent the deafness phenotype from developing in the adult stage. A modified Adeno-associated virus construct carrying a *Kcnq1* expression cassette was injected postnatally (P0-P2) into the endolymph, . …… "

In speculating what it would take to perform success gene therapy in mature ears, we added the following concluding sentences near the end of Discussion (starting from page14 line302): "……Our results in the auditory system suggest that as long as noninvasive gene delivery to the marginal cells in the scala media can be achieved in sufficient quantity and before the degeneration of cochlear cells, the human efficacy of such treatment suggested by the current study is optimistic. Recent advances in developing new viral vehicles aimed at effectively penetrating the blood-brain barrier (Foust et al, 2009; Manfredsson et al, 2009) or diffusing across dense tissue for gene delivery in the eyes (Dalkara et al, 2013) are promising candidates for further examining whether these methods could be used to treat genetic deafness in humans."

The entire first paragraph is dedicated to what was done or not done by others, …… It would be more elegant to be informative about the specific mutation in humans, the mouse, the approach... etc.

The introduction needs more detail about Kcnq1, the gene family, structure, function, gene roles in other tissues, other mutations, and more on the human phenotype.. …… The second paragraph (line 60) jumps from topics, needs focus.

------Answer: We have substantially modified the Introduction to incorporate the Reviewer's suggestions. The relevant sections of the Introduction are copied here (starting from page3 line70):

"……Mutations in the *KCNQ1* gene (also known as *KvLQT1* or *Kv7.1*) are associated with Jervell Lange-Nilsen (JNL) syndrome (Jervell & Lange-Nielsen, 1957), the phenotypes of which include congenital deafness and long QT intervals in the cardiogram, as well as sudden infant death syndrome and Romano-Ward syndrome (Lee et al, 2000). *KCNQ1* is widely expressed in cardiovascular muscle cells, the kidneys and stomach, and marginal cells in the SV of the inner ear. The *KCNQ1* is a voltage-gated potassium channel; its protein has 676 residues. The KCNQ1 consists of a cytosolic N-terminal domain followed by the S1-S4 voltage sensor, a canonical pore (S5-P-S6) domain, and a long cytosolic C-terminus. At least 16 mutations in the *KCNQ1* gene, typically recessive, cause JLN syndrome (Casimiro et al, 2001). The most common ones are missense mutations that result in single amino-acid residue replacements. In the inner ear, *KCNQ1* co-assembles with *KCNE1* to play critical roles in the secretion of K^+ into the endolymph and the establishment of the endocochlear potential (EP) (Lang et al, 2007).

The endolymphatic space in the cochlear duct is bound by epithelial cells of the membranous labyrinth on three sides by the Reissner's membrane, the reticular lamina, and the lateral wall (Fig. 1A). *Kcnq1* is expressed exclusively in the apical membrane of the marginal cells in the SV. SV in the inner ear generates a high concentration of K^+ in the endolymph and high extracellular endocochlear potential (EP, ~+80 mV), both of which are crucial for the transduction of sound by HCs into neural signals (Lang et al, 2007). Inactivating the *Kcnq1* in mice produces a completely deaf model of human JLN syndrome, although the cardiac phenotypes are less prominent (Casimiro et al, 2001; Lee et al, 2000). In this study, we injected a *Kcnq1*-expressing AAV1 viral construct into the endolymph of *Kcnq1*$^{-/-}$ mice in the early postnatal period. Results demonstrated for the first time that a gene therapy approach could be applied in a mouse model of JLN syndrome to successfully treat gene defects specifically affecting functions of the SV. …… ".

Correct this sentence for style: Kcnq1 is exclusively expressed by the marginal cells in the SV, where the high concentration of K+ in the endolymph and high extracellular endolymphatic potential (EP, ~+80 mV) are generated, and both are crucial for the transduction of sound by hair cells into neural signals...

------Answer: We changed this long sentence into two sentences (starting from page3 line85):

"……*Kcnq1* is expressed exclusively in the apical membrane of the marginal cells in the SV. SV in the inner ear generates a high concentration of K^+ in the endolymph and high extracellular endocochlear potential (EP, ~+80 mV), both of which are crucial for the transduction of sound by HCs into neural signals (Lang et al, 2007). ……".

line 71 produced a mouse model

line 76: These results demonstrated for the first time

line 82 expression

------Answer: Changes were made as suggested.

Line 84: the EL space is bordered by epithelial cells of the membranous labyrinth, not by the basilar membrane…… The Results section should not start with Introduction material

------Answer: This sentence (page3 line83) was changed to "The endolymphatic space in the cochlear duct is bound by epithelial cells of the membranous labyrinth on three sides by the Reissner's membrane, the reticular lamina, and the lateral wall (Fig. 1A). *Kcnq1* is expressed exclusively in the apical membrane of the marginal cells in the SV. ……".

As suggested, the introductory sentence originally in the beginning of the Results section is moved to the Introduction (starting from page3 line83).

Fig 1 D - F: re SGN expression, the observation is probably correct, but expression may also possibly be in Schwann cell, ……

------Answer: We avoided the use of "spiral ganglion neurons" in the description here (and also in the Abstract and other relevant descriptions). We described the finding as "cells in the spiral ganglia". The relevant description was changed to (starting from page5 line121): "……we found that *Kcnq1* was detectable not only in the marginal cells in the SV (see arrow, Fig. 1, D & F), but also ectopically in cells in the lateral wall, spiral ganglia (arrowheads, Fig. 1, D & F), and spiral limbus regions (double arrows, Fig. 1, D&F). ……"

Line 126 suggesting signs of cellular degeneration... not sure unless a later stage is done. Clearly, the cell borders and junctions are not normally organized. Also: since phalloidin mostly stains the adherens junction, it is nice for delineating cell-cell borders but does not reveal much about the tight junctions. …… .

------Answer: Connexins are not a component of tight junctions. We have added new data in the Fig. 3E&F to show the disorganization of the highly-organized structure of the marginal cells in the stria vascularis, and changed the description to (starting from page6 line141): "……In the untreated cochleae of *Kcnq1*$^{-/-}$ mice, we observed that the orderly hexagonal organization of marginal cells (Fig. 3, A-C & E) was damaged (Fig. 3F). We also found that, compared to marginal cells in WT mice (Fig. 3E), the sizes of marginal cells in untreated *Kcnq1*$^{-/-}$ mice vary greatly. Moreover, many cells in untreated *Kcnq1*$^{-/-}$ mice had missing nuclei (arrows in Fig. 3E), suggesting cellular degeneration or distress. ……".

Line 134: move discussion items to discussion, ……

A better explanation is needed regarding why GFP is in the cytoplasm and Kcnq1 in the membrane.

------Answer: Discussion items are moved to the Discussion section now.

As regard to why GFP is in the cytoplasm and *Kcnq1* is in the membrane, we provided an explanation in the Discussion (starting from page11 line234): "……Since ectopic *Kcnq1* expression in WT mice did not damage normal hearing (Fig. 5B, data points shown by filled circles), we conclude that *Kcnq1* expressed in the cells in the spiral ganglia, in fibrocytes of the lateral wall and interdental cells (Fig. 1D & F) probably did not form K^+ channels that are harmful to the function of those cells. This is not surprising since it is known that functional potassium channels in the marginal cells need the co-assembly of *Kcnq1* and *Kcne1* (Lang et al, 2007). In addition to the lack of proper intercellular trafficking of *Kcnq1* to the cell membrane we have observed (Fig. 2), the expression of *Kcne1* may be lacking in ectopic cellular locations, thus preventing the formation of membrane channels. "

The changes in Reissner's membrane are striking. …… Has this been described before in any other pathology, genetic or environmental? With such anatomy, if the phenotypic rescue is attempted at P21 and the SV starts to function, would perilymph become endolymph like?

------Answer: The striking phenotype of a collapsed Reissner's membrane is described in the first paper that phenotypes in *Kcnq1*$^{-/-}$ (then called *Kvlqt1*$^{-/-}$) mice are reported (Lee et al, 2000). We have cited this paper. We have changed the description to (starting from page7 line166): "Consistent with previous reports (Casimiro et al, 2001; Lee et al, 2000), we found that untreated *Kcnq1*$^{-/-}$ mice had a collapsed Reissner's membrane, which was adherent to the spiral ligament and the tectorial membrane, resulting in disappearance of the SM. We also observed degeneration of inner and outer HCs, as well as supporting cells in the organ of Corti (compare Fig. 4A & B) and secondary degeneration of cells in the spiral ganglia (Fig. 4B). The mesothelial cells on the scala tympani side of the basilar membrane were absent as well …… ".

One of the important tasks in conducting pre-clinical trials in animal models has always been to find out reasonable boundary conditions for treatment options in humans, assuming that the time course of disease progression and the phenotype characteristics are similar in humans. For example, if similar collapse of Reissner's membrane and degeneration of multiple types of cochlear cells happen in human as the animal model suggests, this may indicate that treatment will need to start to intervene in immature cochlea. Any therapeutic approach designed at a time point after

malformation of the cochlea will be significantly more difficult. This may apply to other deafness genetic mutations that predominantly affect the morphological development of the cochlea. We have added the following in the Discussion (starting from page13 line265): "……Because of the collapse of the Reissner's membrane and degeneration of the multiple types of cochlear cells observed in the mouse model (Fig. 4B), it appeared that the optimal timing for treatment of the *Kcnq1* null mutation would be before these permanent histological changes happen. Any therapy implemented after malformation of the cochlea would be significantly more difficult. This finding may also serve as a guide to the treatment of other genetic deafness mutations that predominantly affect the morphological development of the cochlea. ……".

I also see in Fig. 4B that mesothelial cells in the ST side of the BM are absent. Perhaps it would be useful to point this out. Their absence is reversed with the treatment.

------Answer: This suggestion was incorporated in the revised manuscript (starting from page8 line171): "The mesothelial cells on the scala tympani side of the basilar membrane were absent as well. In treated *Kcnq1*$^{-/-}$ cochleae, the collapse of the Reissner's membrane was prevented (Fig. 4C), as was the death of hair cells, mesothelial cells, and cells in the spiral ganglia (Fig. 4C). ……".

Line 168: the argument about "stable results" is unclear. The take-home message I get from the data is that in this model SM injections are needed for reaching cells of the membranous labyrinth, ……. Also, these results are not in agreement with the Akil data on Vglut3, a fact that could be mentioned and discussed.

------Answer: We have provided a new Table 1 and substantially modified the Discussion comparing many key aspects of the current study and published results by Akil et al (2012). As suggested, a detailed comparison of the two studies is given in the Discussion (starting from page10 line215):

"When this study is compared to this work, important similarities and differences emerge, as summarized in the Table 1. When reviewing this Table one important difference needs to bear in mind is that the cellular targets for treatment in the two studies are hair cells in the organ of Corti and marginal cells in the SV respectively, which are two different sites in the cochlea.

……(Table 1 inserted here). It is interesting to note that although the same AAV subtype and promoter were used in both studies, our injections into the ST generally failed to transduce any cells lining the endolymphatic space (Supplemental Fig. 1). This study also yielded a number of novel findings, among them the extensive ectopic expression shown by virally mediated *Kcnq1* in the cochlea (Figs. 1 & 2). These results are in contrast to virally expressed VGLUT3, which was exclusively in 100% of the inner HCs (Akil et al, 2012). Considering that other virally expressed exogenous proteins, including GFP (Akil et al, 2012; Wang et al, 2013) and connexin26 (Yu et al, 2013), all showed nonspecific expression, our results suggest that the cellular specificity achieved by VGLUT3 driven by a nonspecific promoter is an exception rather than a common phenomenon. The ectopic *Kcnq1* expression demonstrated in this work also gave us an opportunity to investigate possible side effects of such expression outside the targeted cells. Since ectopic *Kcnq1* expression in WT mice did not damage normal hearing (Fig. 5B, data points shown by filled circles), we conclude that *Kcnq1* expressed in the cells in the spiral ganglia, in fibrocytes of the lateral wall and interdental cells (Fig. 1D & F) probably did not form K^+ channels that are harmful to the function of those cells. This is not surprising since it is known that functional potassium channels in the marginal cells need the co-assembly of *Kcnq1* and *Kcne1* (Lang et al, 2007). In addition to the lack of proper intercellular trafficking of *Kcnq1* to the cell membrane we have observed (Fig. 2), the expression of *Kcne1* may be lacking in ectopic cellular locations, thus preventing the formation of membrane channels.

Our results also showed for the first time that virally expressed exogenous protein was correctly trafficked intracellularly to its native membrane location (Fig. 2). *Kcnq1* encodes a potassium channel subunit that is known to be required for generation of the EP and the high K^+ concentration, both of which are essential for auditory transduction (Barhanin et al, 1996; Sanguinetti et al, 1996). We found that exogenous *Kcnq1* was correctly targeted to these apical membranes; this was in sharp contrast to the diffuse intracellular distribution of virally expressed GFP or VGLUT3 in inner HCs (Akil et al, 2012). Interestingly, ectopically expressed connexin 26 (Cx26) in marginal cells is not transported to the cell membrane (Yu et al, 2013), but virally

expressed Cx26 in supporting cells was correctly targeted to the cell membranes to form gap junctions (Yu et al, 2013). These findings suggest that crucial endogenous protein regulatory mechanisms govern the transportation and assembly of virally expressed proteins. These proteins are able to be trafficked like native proteins and co-assembled with their native molecular partners to form functional membrane channels. ".

The Discussion organization, tone and focus need to change.

It now focuses on advances made by the authors and on clinical applicability. In reality, clinical applicability is very remote because current data are on developing ears in "ear age" equivalent to roughly mid-gestation in humans. Also, SM access is currently difficult to impossible, at any age.

…… Instead, I suggest the focus should be on cell biology of KCNq1, its role in the SV, why its presence in other ear cell types does not degrade thresholds, and why hearing is not better than normal and EP not higher than normal when levels of KCNQ1 are increased beyond normal. Discussion of the technique and choice of vector is also interesting and relevant (some of this is included, but some expansion may be useful). Best to conclude with the clinical applicability (or lack thereof) and what it would take to perform this on mature ears and accomplish rescue in the relevant model system and in human.

------Answer: We have substantially modified the Discussion to incorporate these suggestions:

1) For suggestion that "*clinical applicability is very remote because current data are on developing ears in "ear age" equivalent to roughly mid-gestation in humans. Also, SM access is currently difficult to impossible, at any age.*", we have discussed these issues near the end of Discussion (starting from page14 line302): "……Our results in the auditory system suggest that as long as noninvasive gene delivery to the marginal cells in the scala media can be achieved in sufficient quantity and before the degeneration of cochlear cells, the human efficacy of such treatment suggested by the current study is optimistic. Recent advances in developing new viral vehicles aimed at effectively penetrating the blood-brain barrier (Foust et al, 2009; Manfredsson et al, 2009) or diffusing across dense tissue for gene delivery in the eyes (Dalkara et al, 2013) are promising candidates for further examining whether these methods could be used to treat genetic deafness in humans. ".

2) For suggestion that "*I suggest the focus should be on cell biology of KCNq1, its role in the SV, why its presence in other ear cell types does not degrade thresholds, ……*", we have added following in the Discussion (starting from page11 line233): "……Since ectopic *Kcnq1* expression in WT mice did not damage normal hearing (Fig. 5B, data points shown by filled circles), we conclude that *Kcnq1* expressed in the cells in the spiral ganglia, in fibrocytes of the lateral wall and interdental cells (Fig. 1D & F) probably did not form K^+ channels that are harmful to the function of those cells. This is not surprising since it is known that functional potassium channels in the marginal cells need the co-assembly of *Kcnq1* and *Kcne1* (Lang et al, 2007). In addition to the lack of proper intercellular trafficking of *Kcnq1* to the cell membrane we have observed (Fig. 2), the expression of *Kcne1* may be lacking in ectopic cellular locations, thus preventing the formation of membrane channels. ".

   We also added the following in the Discussion to further address this question (starting from page11 line243): "……*Kcnq1* encodes a potassium channel subunit that is known to be required for generation of the EP and the high K^+ concentration, both of which are essential for auditory transduction (Barhanin et al, 1996; Sanguinetti et al, 1996). We found that exogenous *Kcnq1* was correctly targeted to these apical membranes; this was in sharp contrast to the diffuse intracellular distribution of virally expressed GFP or VGLUT3 in inner HCs (Akil et al, 2012). Interestingly, ectopically expressed connexin 26 (Cx26) in marginal cells is not transported to the cell membrane (Yu et al, 2013), but virally expressed Cx26 in supporting cells was correctly targeted to the cell membranes to form gap junctions (Yu et al, 2013). These findings suggest that crucial endogenous protein regulatory mechanisms govern the transportation and assembly of virally expressed proteins. These proteins are able to be trafficked like native proteins and co-assembled with their native molecular partners to form functional membrane channels. " .

The Li citation should probably be Dual et al. (first name Mao Li). I did not check other citations.

------Answer: The error in this citation was corrected as suggested.

Line 298: were authors approved in their animal use protocol to operate on both ears of the P 0-2 mouse? At same session? More details are needed.

------Answer: The description for the injection procedure was expanded. More information was given (page17 line349): "……WT and *Kcnq1*$^{-/-}$ mice were injected in the left endolymphatic space with pAAV1-CB7-*Kcnq1* between postnatal day 0 (i.e., the day they were born, P0) and P2. The contralateral ear of the same mouse, which was used as a control, was either injected with pAAV1-CB7-*EGFP* or given no injection. The choice of viral subtype and the timing of injections at the early postnatal stage were based on our published results (Wang et al, 2013; Yu et al, 2013). The pAAV1-CB7-*Kcnq1* viral construct was confirmed by *in-vitro* transfection of HEK293 cells, which showed that 100% of the cells in cultures were transfected *in vitro*. Each injection took about 10 min to complete. The surgery protocol was approved by the Emory IACUC protocol. " .

We specifically noted that "Each injection took about 10 min to complete. The surgery protocol was approved by the Emory IACUC protocol.".

Histology was done at ages …… It would be helpful to know how many of the animals were processed at the later time points, which would make the rescue outcome more exciting.

------Answer: We have added new data in Fig. 5C showing the long-term treatment effect. We found that, even in *Kcnq1*$^{-/-}$ mice that showed worsening click ABR thresholds later, the gross cochlear morphology of the mice were normal in terms of the morphology of hair cells and cells in the spiral ganglia, and the position of the Reissner's membrane. These new results are given in the revised manuscript (starting from page9 line191): "……We examined the effect of hearing preservation in treated *Kcnq1*$^{-/-}$ mice for up to 30 weeks (Fig. 5C). Click-ABR results (n = 5) demonstrated that the treatment effect was stable for the initial 18 weeks, then started to decline (Fig. 5C) at a rate of about 1.4 dB/week. By the end of 30 weeks, click-ABR thresholds in the treated mice increased by about 17 dB on average. However, the difference between treated and untreated ears was still statistically significant (student t-test, $p < 0.05$). In addition, we found that even in *Kcnq1*$^{-/-}$ mice that had worsening click-ABR thresholds (n = 3, data not shown), the gross cochlear morphology was normal in terms of hair cells and cells in the spiral ganglia, as well as the position of the Reissner's membrane." .

The Impact section should be revised as the rest of the document is revised based on the comments.

------Answer: we have modified the impact section, which is copied here (starting from page21 line449): "……IMPACT: This is the first gene therapy study in which we successfully prevented deafness from developing in the adult stage of a mouse model of JLN syndrome caused by gene defects specifically affecting the function of the SV. Since the SV is a common site affected by many deafness genes, the proof-of-principle results shown here suggest that other genetic mutations (e.g., mutations in *KCNE1*, *CCDC50, DFNA5, MYH14, TFCP2L3* and *TMPRSS3* genes) specifically affecting SV function may also be treated by a similar approach. With improvement of the noninvasive viral delivery method developed for adult-stage cochlea, the therapeutic effectiveness of this type of genetic deafness could, in the future, be examined in humans. ".

Some of the figure legends need to include a little more detail to orient non-experts (in inner ear histology) around the tissue.

------Answer: We have re-written and added more details in the figure legends, as suggested by this Reviewer. These are too numerous to list here.

Referee #2:

……These new strategies are currently on their way to their translation into the clinics. Promising results in the transduction of hair cells have been achieved with adenoviral and adeno-associated viral vectors (AAV). As such, Akil et al. could recently reach a 100% transduction efficiency of the target inner hair cells and successful restoration of the Vglut3 deficiency when using an AAV1 vector. Remarkably, Vglut3 was found to be expressed only in the target cell population, although a non-specific promoter was used to express the transgene

.

------Answer: In the Discussion, we have presented a new Table 1 to compare various aspects of this work with those of Akil (2012). This section of the Discussion (starting from page10 line215) is copied here:

"……When this study is compared to this work, important similarities and differences emerge, as summarized in the Table 1. When reviewing this Table one important difference needs to bear in mind is that the cellular targets for treatment in the two studies are hair cells in the organ of Corti and marginal cells in the SV respectively, which are two different sites in the cochlea.

Table 1. Comparison of the current study and a published study by Akil et al. (Akil et al, 2012)

	**This study**	**Akil et al.**
Viral subtype	AAV1	AAV1
Promoter used	CBA	CBA
Virus injection time	P0-P2	P1-P12
Repeated with different batches of viral solution	Yes (Supplemental Fig. 2)	Unclear
Targeted cells	Marginal cells	Inner HCs
Ectopic expression of therapeutic gene	Yes, and extensive	No
Ectopic expression of GFP	Yes	Yes
Long-term treatment effect	Deteriorated after 18 weeks	Maintained for at least 9 months
Intracellular trafficking	Located to apical membrane of the marginal cells	Stayed uniformly and intracellularly in the inner HCs
% of expression in targeted cells	75 ± 5% , 71 ± 8% and 61 ± 10% for marginal cells in the basal, middle and apical turns, respectively	100% in the inner IHC through the cochlear turns
ST delivery for Trans-scala expression	Generally poor	100% inner HCs were transduced

It is interesting to note that although the same AAV subtype and promoter were used in both studies, our injections into the ST generally failed to transduce any cells lining the endolymphatic space (Supplemental Fig. 1). This study also yielded a number of novel findings, among them the extensive ectopic expression shown by virally mediated *Kcnq1* in the cochlea (Figs. 1 & 2). These results are in contrast to virally expressed VGLUT3, which was exclusively in 100% of the inner HCs (Akil et al, 2012). Considering that other virally expressed exogenous proteins, including GFP (Akil et al, 2012; Wang et al, 2013) and connexin26 (Yu et al, 2013), all showed nonspecific expression, our results suggest that the cellular specificity achieved by VGLUT3 driven by a nonspecific promoter is an exception rather than a common phenomenon. The ectopic *Kcnq1* expression demonstrated in this work also gave us an opportunity to investigate possible side effects of such expression outside the targeted cells. Since ectopic *Kcnq1* expression in WT mice did not

damage normal hearing (Fig. 5B, data points shown by filled circles), we conclude that *Kcnq1* expressed in the cells in the spiral ganglia, in fibrocytes of the lateral wall and interdental cells (Fig. 1D & F) probably did not form K^+ channels that are harmful to the function of those cells. This is not surprising since it is known that functional potassium channels in the marginal cells need the co-assembly of *Kcnq1* and *Kcne1* (Lang et al, 2007). In addition to the lack of proper intercellular trafficking of *Kcnq1* to the cell membrane we have observed (Fig. 2), the expression of *Kcne1* may be lacking in ectopic cellular locations, thus preventing the formation of membrane channels.

Our results also showed for the first time that virally expressed exogenous protein was correctly trafficked intracellularly to its native membrane location (Fig. 2). *Kcnq1* encodes a potassium channel subunit that is known to be required for generation of the EP and the high K^+ concentration, both of which are essential for auditory transduction (Barhanin et al, 1996; Sanguinetti et al, 1996). We found that exogenous *Kcnq1* was correctly targeted to these apical membranes; this was in sharp contrast to the diffuse intracellular distribution of virally expressed GFP or VGLUT3 in inner HCs (Akil et al, 2012). Interestingly, ectopically expressed connexin 26 (Cx26) in marginal cells is not transported to the cell membrane (Yu et al, 2013), but virally expressed Cx26 in supporting cells was correctly targeted to the cell membranes to form gap junctions (Yu et al, 2013). These findings suggest that crucial endogenous protein regulatory mechanisms govern the transportation and assembly of virally expressed proteins. These proteins are able to be trafficked like native proteins and co-assembled with their native molecular partners to form functional membrane channels."

1) Clear presentation of the experimental setup and the related results is not good. This includes:

- size of the different mouse cohorts used ……

- which mouse received which vector dosage? Any differences in the outcome?

- Was the Kcnq1 transgene codon optimized?

……

<u>------Answer:</u> We have substantially modified the Materials and Method section (starting from page16 line343) to address the issues raised by this Reviewer. The added sections (starting from page16 line342) are copied here:

To answer "*size of the different mouse cohorts used ……*":

"……Mice were divided into four groups (N >6): WT controls; WT mice given viral injections into either the scala media or scala tympani; *Kcnq1*$^{-/-}$ mice given viral injection into the scale media and used for ABR and cochlear morphological examinations; and *Kcnq1*$^{-/-}$ mice given viral injection into the scale tympani and used for ABR and cochlear morphological examinations. The specific number of mice in each group is given in the Results. Mice were anesthetized by placing them on ice. An incision was made in the skin behind the ear to expose the otic bulla. The tympanic membrane and auditory ossicles were used as landmarks during surgery. The location of the basal cochlear turn was distinguished by its anatomical relation to the stapedius artery. ……"

To answer "w*hich mouse received which vector dosage? Any differences in the outcome?*":

(starting from page17 line349): "…… WT and *Kcnq1*$^{-/-}$ mice were injected in the left endolymphatic space with pAAV1-CB7-*Kcnq1* between postnatal day 0 (i.e., the day they were born, P0) and P2. The contralateral ear of the same mouse, which was used as a control, was either injected with pAAV1-CB7-*EGFP* or given no injection. The choice of viral subtype and the timing of injections at the early postnatal stage were based on our published results (Wang et al, 2013; Yu et al, 2013). ……"

To answer "*Was the Kcnq1 transgene codon optimized?*":

(starting from page17 line354): "……The pAAV1-CB7-*Kcnq1* viral construct was confirmed by *in-vitro* transfection of HEK293 cells, which showed that 100% of the cells in cultures were transfected *in vitro*. ……".

2) The main point/advance of the presented data is the correction of genetic defects that affect the stria vascularis (SV). Repetitive experiments to prove the reliability of the obtained results is

© EMBO

therefore decisive. Thus, it is unclear why the authors do not show the important 'negative' results of their injections in the scala tympani (ST) through the round window membrane and associated repetitions for SV and ST injections performed by different researchers or, better said, independent experiments, respectively (page 7, lines 163 - 168).

------Answer: We have presented "negative" results of injections made into the scala tympani in the new Supplemental Fig. 2B. Repetitions injection results, as obtained by different researchers (presented as different symbols), are also given in the new Supplemental Fig. 2A.

3) There are kind of diverging results by the recent Akil et al. study and the data presented here (page 10, lines 225 - 231). Please discuss this aspect in more detail, including AAV serotypes and dosages used, animal age at infection and time of sacrifice etc.

------Answer: We have given a new Table 1 to compare the details of this and Akil studies. This question was answered in detail in answering the general comments of this Reviewer.

4) Very important, as the authors outline in their last paragraph of the discussion section, is the longterm effect of hearing restoration beyond the 1 -3 months study points …… at least to give a hint whether efficiency without side effects remains stable?

------Answer: We have given new data in Fig. 5C of revised manuscript to show the longer-term effect, up to 30 weeks following the treatment, as measured by click ABR. The relevant descriptions (starting from page9 line193) are copied here:

"……We examined the effect of hearing preservation in treated *Kcnq1*$^{-/-}$ mice for up to 30 weeks (Fig. 5C). Click-ABR results (n = 5) demonstrated that the treatment effect was stable for the initial 18 weeks, then started to decline (Fig. 5C) at a rate of about 1.4 dB/week. By the end of 30 weeks, click-ABR thresholds in the treated mice increased by about 17 dB on average. However, the difference between treated and untreated ears was still statistically significant (student t-test, $p < 0.05$). In addition, we found that even in *Kcnq1*$^{-/-}$ mice that had worsening click-ABR thresholds (n = 3, data not shown), the gross cochlear morphology was normal in terms of hair cells and cells in the spiral ganglia, as well as the position of the Reissner's membrane. ".

5) The authors should comment on the clinical translation opportunity of their early postnatal approach. This approach will not work for adult mice and for humans, right? The route to go will then be directly via the perilymph, rather than the scala media?

------Answer: We have added following comments in the Discussion (starting from page12 line255): "One of the important tasks in conducting preclinical trials in animal models, assuming that the time course of disease progression and the phenotype characteristics observed in animal models can be applied in humans, is to examine reasonable boundary conditions for optimal treatment options in humans. ……" For examples, if the genetic mutation mainly affect the morphological development of the cochlear, then it is unlikely that treatment performed at adult stage would be successful due the malformation of the auditory organ. In the case of *Kcnq1*$^{-/-}$ mice, scala media and endolymph disappear at the adult stage, Reissner's membrane collapses, cells in the organ of Corti and spiral ganglia degenerate. These disease phenotypes and the hearing preservation results demonstrated in this paper suggest that treatment performed at the early postnatal stage (in mice) is the optimal time (although inconvenient for human translation) to intervene for this particular genetic condition.

Based on the results of this study, we further listed a few boundary conditions for treatment of Kcnq1 null mutations in the Discussion (starting from page12 line260): "……(1) We found that in order to have the hearing preserved in the *Kcnq1*$^{-/-}$ mice, the percentage of marginal cells expressing the *Kcnq1* need not to be 100%. By immunolabeling criteria, the percentage of marginal cells expressed *Kcnq1* after viral injections is in the range of 61%-75% (Fig. 3F). Whether higher transduction efficacy may give better or longer-lasting treatment effect is unknown.

(2) Because of the collapse of the Reissner's membrane and degeneration of the multiple types of cochlear cells observed in the mouse model (Fig. 4B), it appeared that the optimal timing for treatment of the *Kcnq1* null mutation would be before these permanent histological changes happen. Any therapy implemented after malformation of the cochlea would be significantly more difficult.

This finding may also serve as a guide to the treatment of other genetic deafness mutations that predominantly affect the morphological development of the cochlea.

(3) With the viral type and promoter we tested in this study, it appears that the results of one-time treatment for mutations affecting the function of SV are not permanent (Fig. 5C). Thus, either a new viral type must be tested or supplemental treatment be done for long-term efficacy. ".

Near the end of Discussion we also added following points to address human translation issues (starting from page14 line299): "……Gene augmentation or replacement therapy for multiple inherited retinal degeneration diseases (e.g., Leber congenital amaurosis, choroideremia, Stargardt's disease, and retinoschisis) has advanced to clinical phase I or phase II trials (Dalkara & Sahel, 2014; Smith et al, 2012). Our results in the auditory system suggest that as long as noninvasive gene delivery to the marginal cells in the scala media can be achieved in sufficient quantity and before the degeneration of cochlear cells, the human efficacy of such treatment suggested by the current study is optimistic. Recent advances in developing new viral vehicles aimed at effectively penetrating the blood-brain barrier (Foust et al, 2009; Manfredsson et al, 2009) or diffusing across dense tissue for gene delivery in the eyes (Dalkara et al, 2013) are promising candidates for further examining whether these methods could be used to treat genetic deafness in humans. ".

6) Each abbreviation has to be tendered before use. Please recheck your manuscript, …… .

------Answer: We have rechecked the first use of all the abbreviations. We have added an abbreviation list before the Reference (staring from page22 line467). It is given here:

4',6-diamidino-2-phenylindole (DAPI)

Adeno-associated virus (AAV)

age-dependent hearing loss (ADHL)

auditory brainstem response (ABR)

chicken β-actin (CBA)

connexin (Cx26)

endolymphatic potential (EP)

green fluorescent protein (GFP)

hair cells (HCs)

Jervell and Lange-Nielsen (JLN)

knockout (KO)

round window (RW)

scala media (SM)

scala tympani (ST)

spiral ganglion neurons (SGNs)

stria vascularis (SV)

wide-type (WT)

Note: QT (as in long-QT) appears to be an accepted term, not an abbreviation. The condition is so named because of the appearances of the electrocardiogram on which a prolongation of the QT interval occurs.

Referee #3:

…… The only ethical issue is the amount of surgery required to inject the cochlea, which is not described in the current text at all.

------Answer: We have given more details about the injection procedure and stated specifically "the surgery protocol was approved in the Emory IACUC protocol".

The relevant sections are copied here (starting from page16line349): "……WT and *Kcnq1*$^{-/-}$ mice were injected in the left endolymphatic space with pAAV1-CB7-*Kcnq1* between postnatal day 0 (i.e., the day they were born, P0) and P2. The contralateral ear of the same mouse, which was used as a control, was either injected with pAAV1-CB7-*EGFP* or given no injection. The choice of viral subtype and the timing of injections at the early postnatal stage were based on our published results (Wang et al, 2013; Yu et al, 2013). The pAAV1-CB7-*Kcnq1* viral construct was confirmed by *in-vitro* transfection of HEK293 cells, which showed that 100% of the cells in cultures were transfected *in vitro*. Each injection took about 10 min to complete. The surgery protocol was approved by the Emory IACUC protocol." .

…… The limitation of the findings is that the injection was carried out at a very immature stage of development,…… the finding suggests that the pathology can be avoided before it begins by transfection with the AAV, but not that the deafness can be treated using this approach. Nonetheless, this is an important and interesting report ……

------Answer: Throughout the revised manuscript we have described that our gene therapy avoided the development of deafness phenotype in *Kcnq1*$^{-/-}$ mice, not that deafness was treated successfully.

Title, abstract, and throughout the text: the human disease is Jervell and Lange-Nielsen, not Javell.

------Answer: This was corrected as suggested throughout the manuscript.

Abstract, line 26: The gene therapy does not treat the deafness phenotype, but instead prevents deafness from developing, so this sentence needs revising.

------Answer: the sentence in the abstract (page1 line25) was changed to "……We used the gene therapy tested in a mouse model of JLN syndrome (*Kcnq1*$^{-/-}$ mice) to prevent the deafness phenotype from developing in the adult stage. ……".

Abstract line 32: The potential is strictly the endocochlear potential, not endolymphatic potential, because not all endolymph-filled chambers have this high potential.

------Answer: The use of "endolymphatic potential " was changed to "endocochlear potential" throughout the manuscript.

Introduction, line 74: The treatment does not restore normal cochlear morphology and function, but instead prevents the pathology from developing, so this sentence needs to be revised.

------Answer: This sentence was a description of the Results, and we have removed it from the Introduction. We have made changes, as suggested, through the manuscript to reflect that we only prevented the deafness phenotype from developing in the *Kcnq1*$^{-/-}$ mice.

Results, line 84: The physiological boundary of scala media and endolymph is the reticular lamina - the upper surface of the hair cells and surrounding supporting cells, not the basilar membrane which is relatively leaky.

------Answer: As suggested, this sentence was changed (page3 line83) to "The endolymphatic space in the cochlear duct is bound by epithelial cells of the membranous labyrinth on three sides by the Reissner's membrane, the reticular lamina, and the lateral wall (Fig. 1A). ……"

© EMBO

Results, line 93: Define SM and ST when first used.

------Answer: SM was defined previously in the subtitle of the Results section (line99). ST is defined here, as suggested.

Results; line 104-105: Say what this comparison reveals, rather than leave it to the reader to guess what the difference is.

------Answer: We modified this sentence (starting from page5 line118) to: "The fact that native *Kcnq1* is found only at the apical membrane of marginal cells (Fig. 1B) (Lang et al, 2007) also facilitated our studies of virally-mediated ectopic *Kcnq1* gene expression. When we compared immunolabeling results obtained in the injected cochlea of WT (Fig. 1D) and *Kcnq1*$^{-/-}$ (Fig. 1F) mice, we found that *Kcnq1* was detectable not only in the marginal cells in the SV (see arrow, Fig. 1, D & F), but also ectopically in cells in the lateral wall, spiral ganglia (arrowheads, Fig. 1, D & F), and spiral limbus regions (double arrows, Fig. 1, D&F). ……"

Results, line 134: In point (1), clarify whether you refer to native Kcnq1 expression, or expression from the transgene.

------Answer: The description was changed to (page7 line155): "……(1) As in cochlea of treated *Kcnq1*$^{-/-}$ mice, native *Kcnq1* expression in a subgroup of marginal cells was always below the detection limit of immunolabeling in WT animals (Fig. 3A). The percentage of *Kcnq1* expression in marginal cells was never 100% in both groups. ……"

Results, line 149: There is no evidence presented that Reissner's membrane has returned to its WT position, …… that the collapse of this membrane was avoided, so this needs rewording.

------Answer: We have changed the description (page8 line172) to "……In treated *Kcnq1*$^{-/-}$ cochleae, the collapse of the Reissner's membrane was prevented (Fig. 4C), as was the death of hair cells, mesothelial cells, and cells in the spiral ganglia (Fig. 4C). ……".

Discussion, line 215: This is the first time that the timing of the injection has been specified, so it would be helpful to the readers to give the stage of injection much earlier in the main text to prevent misunderstanding.

------Answer: We have given the timing of injection in the abstract of the modified manuscript (page1 line27) "A modified Adeno-associated virus construct carrying a *Kcnq1* expression cassette was injected postnatally (P0-P2) into the endolymph, which resulted in *Kcnq1* expression in most cochlear marginal cells where the native *Kcnq1* is exclusively expressed. ……". More details are given in the Method section (starting from page17 line349): "……WT and *Kcnq1*$^{-/-}$ mice were injected in the left endolymphatic space with pAAV1-CB7-*Kcnq1* between postnatal day 0 (i.e., the day they were born, P0) and P2. "

Discussion, lines 236-237: Even though the surgical technique has been described in a previous paper, it would be very helpful to give a brief outline in this paper (either here or in the methods) so that the implications for gene therapy can more easily be appreciated by the readers.

------Answer: We have added more details in the Materials and Methods section about the surgical procedures (starting from page16 line346): "Mice were anesthetized by placing them on ice. An incision was made in the skin behind the ear to expose the otic bulla. The tympanic membrane and auditory ossicles were used as landmarks during surgery. The location of the basal cochlear turn was distinguished by its anatomical relation to the stapedius artery. WT and *Kcnq1*$^{-/-}$ mice were injected in the left endolymphatic space with pAAV1-CB7-*Kcnq1* between postnatal day 0 (i.e., the day they were born, P0) and P2. The contralateral ear of the same mouse, which was used as a control, was either injected with pAAV1-CB7-*EGFP* or given no injection. The choice of viral subtype and the timing of injections at the early postnatal stage were based on our published results (Wang et al, 2013; Yu et al, 2013). The pAAV1-CB7-*Kcnq1* viral construct was confirmed by *in-vitro* transfection of HEK293 cells, which showed that 100% of the cells in cultures were transfected

© EMBO

in vitro. Each injection took about 10 min to complete. The surgery protocol was approved by the Emory IACUC protocol.

Viruses used in injections were resuspended in 0.01 M phosphate buffer. Injection of a small amount of fluid was done using a Picospritzer III pressure microinjection system (Picospritzer III; Parker Hannifin, NY). The pressure source was an air tank regulated at an output pressure of 20 psi. Glass micropipettes with a tip size of 10-15 µm were made on a P-2000 horizontal pipette puller (Sutter Instrument, Novato, CA), back filled with viral solution, and controlled by a micromanipulator (MP-285, Sutter Instrument, Novato, CA). The glass micropipettes were controlled to penetrate into either the scala media through the soft bony cochlear shell of early postnatal mice near the basal cochlear turn or into the scala tympani through the round window membrane. We ejected approximately 0.5 µl of fluid out of the tip of glass pipettes by controlling the duration (12 msec) and the number of pressure pulses (set at 12). Fast green dye (Sigma-Aldrich, St Louis, MO), which is visible under bright-field illumination with a dissecting microscope (Stemi2000; Carl Zeiss, Oberkochen, Germany), was included in the solution to help visually confirm fluid ejection. After surgery, mice were allowed to recover on a 37 °C heating pad (model TR-100, Fine Science Tool Inc., Foster City, CA) before returning to the animal housing facility. More details of surgical and injection procedures have been given previously (Wang et al, 2013; Yu et al, 2013). ……"

Discussion, line 259: The data for three different batches of viral solutions was not presented, so this conclusion is not supported by the results presented. Maybe the ABR thresholds for individual mice could be presented with batch number indicate by different symbols in the ABR threshold figure?

------Answer: As suggested, we have added a new Supplemental Figure 2. ABR thresholds obtained by different experimenters were given in different symbols. Following description was added in the revised manuscript (starting from page8 line189): "……We have repeated viral inoculations using solutions independently made from three batches. Data obtained by four different experimenters showed essentially the same results, suggesting that the treatment protocol used in this study yielded stable results (Supplemental Fig. 2A).".

Legend for supplemental fig. 2 (starting from page30 line682) is also copied here: "(A) Unaveraged ABR thresholds measured from individual animals. Different symbols represent results obtained by four different experimenters. Filled symbols are ABR results obtained from untreated *Kcnq1*$^{-/-}$ mice. (B) ABR thresholds obtained from *Kcnq1*$^{-/-}$ mice received viral injection into the scala tympani."

Methods, line 303-304: Say briefly what the exposure method involved.

------Answer: This question was answered previously. We have substantially added more details for the surgery procedure. Relevant Method sections (starting from page16 line346) are copied here: "……An incision was made in the skin behind the ear to expose the otic bulla. The tympanic membrane and auditory ossicles were used as landmarks during surgery. The location of the basal cochlear turn was distinguished by its anatomical relation to the stapedius artery. WT and *Kcnq1*$^{-/-}$ mice were injected in the left endolymphatic space with pAAV1-CB7-*Kcnq1* between postnatal day 0 (i.e., the day they were born, P0) and P2. The contralateral ear of the same mouse, which was used as a control, was either injected with pAAV1-CB7-*EGFP* or given no injection. ".

Methods, line 305: Was artificial endolymph used when injecting into scala tympani, rather than artificial perilymph?

------Answer: We used 0.01 M phosphate buffer. At the early postnatal stage, high K^+ and endocochlear potential are developed yet. The description (starting from page17 line358) is copied here: "Viruses used in injections were resuspended in 0.01 M phosphate buffer. Injection of a small amount of fluid was done using a Picospritzer III pressure microinjection system (Picospritzer III; Parker Hannifin, NY). ……"

Figures: It appears that the scale bars are incorrect (too short for the distance given) for all figures. Please check.

------Answer: We have checked the scale bars in the original image data. Corrections were made whenever necessary in the figures to reflect the correct scale of the figures.

Following changes were made in the text to reflect the changes we made in figures:

Page27 line615: Legend for Figure 1 "Scale bars represent approximately 200 µm in all panels." was changed to "Scale bars represent approximately 100 µm in all panels.".

Page28 line630: Legend for Figure 2 "Scale bars represent appropriately 100 µm in all panels." was changed to "Scale bars represent appropriately 50 µm in all panels."

Page28 line642: Legend for Figure 3 "Scale bars represent approximately 100 µm" was changed to "Scale bars represent approximately 50 µm in panels A-D."

Page29 line647: Legend for Figure 4 "Scale bars represent approximately 100 µm." was changed to "Scale bars represent approximately 50 µm.".

Fig 2 legend, B, line 518: Clarify whether this mouse was injected or not.

------Answer: Changes were made as suggested. Legend for Figure 2B (starting from page28 line623) was changed to "……B) Cryosection through the SV of a *Kcnq1*$^{-/-}$ mouse injected with AAV expressing *Kcnq1*. ", in order to indicate that this mouse was injected.

Fig 3 legend, line 528 and 529: These appear to be whole mounts not sections, so remove the words "section".

------Answer: Changes were made as suggested. The word "section" was removed from the legend of Figure 3.

Expanded view, Fig 1: Is this a supplementary figure? It does not look like an expanded view of Fig 1. State whether the samples are from mutants or WT mice.

------Answer: This is supplementary figure 1. It is not the expanded view of Fig. 1 in the main text. The cochlear sections were obtained from the WT mice, and we added following text in the figure legend (page30 line664) to clarify this: "Virally mediated GFP expression in cochlea obtained by two different injection routes. (A) Results obtained by injection into the SM and (B) ST of WT mice are compared. …… ".

2nd Editorial Decision 15 May 2015

Thank you for the submission of your revised manuscript to EMBO Molecular Medicine. Unfortunately Reviewer #1 was not available to re-evaluate your revised manuscript. However, I asked the Reviewers to also verify whether you had also satisfactorily addressed Reviewer 1's concerns.

We have now received the enclosed reports from the Reviewers that were asked to re-assess it. As you will see they are now globally supportive and I am pleased to inform you that we will be able to accept your manuscript pending the following final issues.

1) I have performed some copyediting (Title, Abstract and The Paper Explained sections) on your manuscript to improve impact and readability. Please find attached a copy of your manuscript highlighting the changes including a couple of comments for your attention/action. Please read carefully and apply any further changes using the attached copy of the manuscript. May I also ask you to carefully go over the entire manuscript to correct some English usage and readability issues?
2) You are welcome to suggest a striking image or visual abstract to illustrate your article online. If you do please provide a high quality jpeg file 550 px-wide x 400-px high.

© EMBO

I look forward to reading a new revised version of your manuscript as soon as possible so that we can proceed with formal acceptance.

***** Reviewer's comments *****

Referee #2 (Comments on Novelty/Model System):

The authors have addressed my critics. The additional re-structuring of the various sections has substantially improved the manuscript.

Referee #2 (Remarks):

All my critics have been adequately addressed.

Referee #3 (Remarks)

The manuscript Is suitable for publication

2nd Revision - authors' response 17 May 2015

I am happy to learn that our paper (EMM-2014-04929-V2) has received positive reviews and potentially is acceptable by your journal. Here are my answers to your questions/comments:

(1) We have accepted your editing in the copy of the manuscript attached with your last email. I have also gone over the manuscript and made a few minor changes in order to enhance readability. The revisions made could be viewed using the Review function of the Microsoft Word.

The manuscript was proofread by a professional Editor, Dr. Jeanne Cole, editor of BIOMEDICAL SCIENCES, for the correct English usage. The new manuscript (EMM-2014-04929-V3) was submitted online from your website.

(2) I am happy to suggest images. Must the image be in the original format (Figs. 1-5), or could we use some of the image panels and remove the arrows and labels? Could we also suggest images in the supplemental materials?

Thanks.
